# Life events and treatment prognosis for depression: A systematic review and individual patient data meta-analysis

**DOI:** 10.1016/j.jad.2021.12.030

**Published:** 2022-02-15

**Authors:** Joshua E.J. Buckman, Rob Saunders, Laura-Louise Arundell, Iyinoluwa D. Oshinowo, Zachary D. Cohen, Ciaran O'Driscoll, Phoebe Barnett, Joshua Stott, Gareth Ambler, Simon Gilbody, Steven D. Hollon, Tony Kendrick, Edward Watkins, Thalia C. Eley, Megan Skelton, Nicola Wiles, David Kessler, Robert J. DeRubeis, Glyn Lewis, Stephen Pilling

**Affiliations:** a Centre for Outcomes Research and Effectiveness (CORE), Research Department of Clinical, Educational & Health Psychology, University College London, London WC1E 7HB, United Kingdom; b iCope – Camden & Islington Psychological Therapies Services, Camden & Islington NHS Foundation Trust, 4St Pancras Way, London NW1 0PE, United Kingdom; c Department of Psychiatry, University of California, Los Angeles, Los Angeles, CA 90095, United States; d Statistical Science, University College London, London WC1E 7HB, United Kingdom; e Department of Health Sciences, University of York, York YO10 5DD, United Kingdom; f Department of Psychology, Vanderbilt University, Nashville, TN 407817, United States; g Primary Care, Population Sciences and Medical Education, Faculty of Medicine, University of Southampton, Southampton SO16 5ST, United Kingdom; h Department of Psychology, University of Exeter, Exeter, EX4 4QG, United Kingdom; i Social, Genetic and Developmental Psychiatry Centre, Institute of Psychiatry, Psychology & Neuroscience, King's College London, London SE5 8AF, United Kingdom; j Centre for Academic Mental Health, Population Health Sciences, Bristol Medical School, University of Bristol, Oakfield House, Bristol, United Kingdom; k Centre for Academic Primary Care, Population Health Sciences, Bristol Medical School, University of Bristol, Canynge Hall, Bristol, United Kingdom; l School of Arts and Sciences, Department of Psychology, University of Pennsylvania, Philadelphia, PA 19104-60185, United States; m Division of Psychiatry, University College London, London W1T 7NF, United Kingdom; n Camden and Islington NHS Foundation Trust, St Pancras Hospital, 4St Pancras Way, London NW1 0PE United Kingdom

**Keywords:** Depression, Treatment outcome, Stressful life events, Individual patient data meta-analysis, Systematic review

## Abstract

**Objective:**

To investigate associations between major life events and prognosis independent of treatment type: (1) after adjusting for clinical prognostic factors and socio-demographics; (2) amongst patients with depressive episodes at least six-months long; and (3) patients with a first life-time depressive episode.

**Methods:**

Six RCTs of adults seeking treatment for depression in primary care met eligibility criteria, individual patient data (IPD) were collated from all six (*n* = 2858). Participants were randomized to any treatment and completed the same baseline assessment of life events, demographics and clinical prognostic factors. Two-stage random effects meta-analyses were conducted.

**Results:**

Reporting any major life events was associated with poorer prognosis regardless of treatment type. Controlling for baseline clinical factors, socio-demographics and social support resulted in minimal residual evidence of associations between life events and treatment prognosis. However, removing factors that might mediate the relationships between life events and outcomes reporting: arguments/disputes, problem debt, violent crime, losing one's job, and three or more life events were associated with considerably worse prognoses (percentage difference in 3–4 months depressive symptoms compared to no reported life events =30.3%(95%CI: 18.4–43.3)).

**Conclusions:**

Assessing for clinical prognostic factors, social support, and socio-demographics is likely to be more informative for prognosis than assessing self-reported recent major life events. However, clinicians might find it useful to ask about such events, and if they are still affecting the patient, consider interventions to tackle problems related to those events (e.g. employment support, mediation, or debt advice). Further investigations of the efficacy of such interventions will be important.

## Highlights

• Depressed patients reporting severely stressful life events had worse prognoses.
• Reporting three or more events was associated with considerably worse outcomes.
• This held in patients with long durations and those in a first depressive episode.
• Effects were attenuated by variables that might have been affected by the events.
• Clinicians should routinely ask patients about life events and assess their impact.

## 1 Introduction

Stressful major life events, such as losing one's job, problematic debt, or divorce, are common (Costello, 1982; McCraw and Parker, 2017; McLaughlin et al., 2010). It is well established that first episodes of depression are often preceded by these kinds of severely stressful experiences (Hammen, 2018; Monroe and Harkness, 2005), and although it is less common for such events to precede subsequent episodes of depression, those who experience major life events are at greater risk of relapse or recurrence (Monroe et al., 2019). The COVID-19 pandemic and related governmental responses are thought to have resulted in an increase in people experiencing many kinds of major life events, such as serious illness (WHO, 2020; Xiao and Torok, 2020), bereavement (Gunnell et al., 2020), losing one's job, and grave financial problems (Gangopadhyaya and Garrett, 2020; Gunnell et al., 2020; Holmes et al., 2020; Xiao and Torok, 2020). They have also been reported to have led to greater exposure of some populations to assault, or other forms of violence (Gunnell et al., 2020; Hall et al., 2020; Takian et al., 2020). More generally, it has been suggested that these kinds of major life events should be measured routinely in clinical practice to inform the management of depression (Weissman et al., 2020).

Although there is a consensus that major life events commonly precede the onset of depression, the role of such experiences post onset is less certain. Of key clinical importance, it is not yet known how these kinds of life events might affect the clinical course and prognosis of patients seeking treatment for depression in general. Further, it is not known if prognostic associations may be clinically pertinent for two important depressed subgroups: those with longer durations of depression (which may include those that were depressed prior experiencing a reported life event) (Lorenzo-Luaces et al., 2020), and those presenting for treatment with a first life-time depressive episode. This latter group of patients has an approximately equal risk of having no future depressive episodes as they do of having a recurrent episode (Monroe et al., 2019). As treatment outcomes are a particularly strong indicator of the risk of future episodes in general (Buckman et al., 2018; Fava et al., 2004), and the plan of treatment for first onset cases is typically quite different from those with a history of recurrences (for whom there is a trend towards indefinite treatment with antidepressants; (Thase, 2006)), advancing knowledge about any prognostic association between major life events and depression treatment outcomes may be particularly valuable.

In an earlier study (Buckman et al., 2021a), we reviewed systematic reviews that reported on associations between patient characteristics and prognosis for adults with depression, in relation to both the natural course of depression for those not treated, and treatment outcomes for those that received any treatment for depression (see Supplementary Table 1). We found only two reviews that reported on associations between life events and prognosis (Paykel, 1994; Steinert et al., 2014). One review was based upon just a single primary study (*N* = 347) (Steinert et al., 2014), and reported no association between life events and prognosis unless participants also lacked social support (Dowrick et al., 2011). It is unclear whether or not the second review was conducted systematically, as limited methodological information was provided (Paykel, 1994). That review addressed both the association of life events and social support in the course of depression for psychiatrically treated patients, employing either general population controls or psychiatric patients with other disorders as controls, 29 studies were included. Some evidence was reported for an association between experiencing life events prior to treatment and poorer course of depression (Paykel, 1994). However, many of the included studies had very small samples (e.g., *n* = 30 (Paykel and Tanner, 1976)) and included adults with other mental health disorders (e.g. schizophrenia, or those with a recent suicide attempt). In contrast, a recent Danish case-register study of 301 adults with major depression reported that neither the experience of any stressful life events prior to the onset of depression, nor the total number of life events experienced before onset was associated with remission from either first or second line antidepressant medications (Bock et al., 2009). Similarly, in a randomised controlled trial of 60 patients receiving cognitive therapy and 120 receiving antidepressant medications, life events (which may have occurred prior or post onset of depression) were not found to be associated with prognosis for either treatment (Fournier et al., 2009). However, a RCT in which 40 participants reported a stressful life event prior to treatment found that this was associated with worse outcomes in the one-third of participants randomized to receive antidepressants, but not in those randomized to either cognitive behaviour therapy or interpersonal psychotherapy (IPT) (Bulmash et al., 2009). Another small study (*n* = 91) found that reporting life events prior to, or during treatment with antidepressants (initially, as an acute-phase treatment) and then IPT (as a continuation-phase treatment) was associated with poorer outcomes for adults with recurrent depression (Monroe et al., 1992). A further prospective study of adults with recurrent depression reported that stressful life events were associated with worse treatment outcomes if patients were considered to have not developed many cognitive skills during such treatment (Vittengl et al., 2020). In addition, a brief non-systematic review has suggested that life events could be important determinants of prognosis for depressed patients (Weissman et al., 2020), but with limited evidence.

Based on these reviews and individual studies, little consistent and reliable evidence exists regarding associations between major life events and treatment outcomes for adults with depression. Further, should an association exist, it remains unclear whether it is general or (1) specific to particular types of life events, (2) relevant regardless of the type of treatment, (3) applicable to distinct subtypes of depressed patients, or (4) unique to life events (as opposed to other prognostic factors).

As there are many types of treatments commonly available for those seeking treatment for depression, it is especially important to determine whether there is a broad prognostic association with life events in general, or with specific types of life events, and treatment outcomes i.e., regardless of the type of treatment received (Buckman et al., 2021a, 2021b). To date, no studies have addressed this matter (Buckman et al., 2021a), instead focussing on prognosis with one particular treatment type only (Bock et al., 2009; Bulmash et al., 2009; Fournier et al., 2009; Monroe and Harkness, 2005; Vittengl et al., 2020), including studies of several treatments in which effects have been assessed within treatment groups. Such studies therefore inform prognosis if the type of treatment due to be received is known at the point a patient presents for assessment, but are not particularly informative if there are a number of treatment options available and the choice of treatment is not determined prior to the assessment (Buckman et al., 2020, 2021a). As this is typically the case in primary care, and large proportions of patients initially present for assessments or treatment in primary care (McManus et al., 2016, Thornicroft et al., 2017), determining associations between major life events and treatment outcomes regardless of treatment type, in a primary care setting, would have clear clinical value.

In addition, few prior studies considered associations of life events with treatment outcomes independent of other prognostic factors that are routinely collected in clinical practice (Buckman et al., 2021a, 2021b). Consequently, it is not known whether there is any incremental prognostic benefit from assessing for life events upon treatment entry. Further, as consultations in primary care are typically very brief (Irving et al., 2017), it is important to evaluate the incremental value of assessing major life events given other potentially informative prognostic factors. More generally, examining associations with prognosis in this way may provide clinicians and patients with useful clinical information about patients’ life circumstances before a choice of treatments has been made (Hippisley-Cox et al., 2007; Trusheim et al., 2007).

The primary aim of this study was therefore to investigate whether the total number of reported major life events, having reported any major life event, and reporting specific major life events in the six months prior to seeking treatment for depression in primary care are associated with treatment prognosis. We aimed to investigate associations independent of treatment type, and independent of markers of depressive severity that we have found to be independently associated with prognosis in a previous study (i.e., the severity of depressive symptoms, the duration of depression, the duration of anxiety concerns, comorbid panic disorder, and a history of antidepressant treatment) (Buckman et al., 2021a). As a secondary aim, to further test the robustness of potential associations, we also sought to investigate effects in two important clinical subgroups of patients common in primary care, that have not been addressed in prior studies: (i) those with depressive episodes whose onset predated the reported life events (rather than those who reportedly experienced stressful life events prior to the onset of their depression) and (ii) those presenting with a first life-time depressive episode.

## 2 Methods & materials

This systematic review with IPD meta-analysis is reported in accordance with the PRISMA-IPD statement (Stewart et al., 2015), see the supplementary materials for the PRISMA-IPD checklist. A general protocol for the formation of the IPD dataset and pre-registered methods for identifying studies are also available (PROSPERO: CRD42019129512 (01/04/2019)). These were reported in line with the PRISMA-Protocol statement (Shamseer et al., 2015) and PRISMA-S (Rethlefsen et al., 2021).

### 2.1 Identification and selection of studies

Studies were identified and selected based on the study protocol (Buckman et al., 2020): by searching Medline, Embase, International Pharmaceutical Abstracts, PsycINFO and Cochrane Central (from inception to October 8th 2021), hand-searching of reference lists, and contacting experts for unpublished or missed studies. No filters or limits were applied to the searches.

At the outset of this project we conducted some preliminary or scoping searches from which it became clear that the Revised Clinical Interview Schedule (CIS-R) (Lewis et al., 1992) was the most commonly used comprehensive measure of depressive and anxiety symptoms, durations and diagnoses, in depression RCTs in primary care (Buckman et al., 2020). In those preliminary searches ten studies used the CIS-R at baseline to determine diagnosis (seven that were published and three protocols for ongoing trials), but only two RCTs used other full comprehensive measures and would likely have met our other inclusion criteria (one used the Schedules for Clinical Assessment in Neuropsychiatry (SCAN) (Wing et al., 1990; Perroud et al., 2012), and one used the full Structured Clinical Interview for DSM (SCID) (Williams et al., 1992; Hegerl et al., 2010)) but neither reported using any measure of life events. The CIS-R was therefore made an inclusion criterion for two reasons: to minimize bias in harmonising data across RCTs (Lesko et al., 2018), and to ensure included studies have data on a range of additional clinical prognostic factors (depressive ‘disorder characteristics’) that can be routinely assessed in clinic. This allowed us to ascertain if reported life events are incrementally informative of prognosis above and beyond such clinical factors. Search terms included variations of phrases such as “depression” or “major depression”, “RCT” or “Randomised Controlled Trial”, and “CIS-R” or “Clinical Interview Schedule” (full details are in Supplementary Table 2).

A single reviewer (JB) screened titles and abstracts of potentially eligible studies, these were then read in full and judged against inclusion/exclusion criteria by two reviewers (JB and GL) with consultation with a third (SP) to resolve uncertainties by consensus.

#### 2.1.1 Inclusion & exclusion criteria

Studies were included if they: were RCTs of adults (aged 16 or over) with unipolar depression, or with depressive symptoms significant enough for them to seek treatment, or a Revised Clinical Interview Schedule (CIS-R) score of ≥12 (the cut-off for common mental disorder) (Lewis et al., 1992); were recruited from primary care; and measured life events that occurred up to six months prior to baseline.

Studies were excluded if they: included patients with depression secondary to personality disorders, psychotic conditions, or neurological conditions; were studies of adults with bi-polar or psychotic depressions, children or adolescents; or were feasibility studies.

Details of the included studies are in Table 1.

**Table 1:** Description of included studies.

**Study**	**N**	**Inclusion criteria**	**Age**	**Gender**	**T0 Depressive Symptom Severity**	**T0 CISR-Total Score**	**T0 Life events Total Score**	**Remission**	**Interventions**	**Depressive Symptom Outcome Measure at 3–4 months**
			Mean (SD)	% Female	Mean(SD)	Mean(SD)	Mean(SD)			
COBALT (1)	469	Adults 18–75 with treatment resistant depression, scoring ≥14 BDI-II	49.6(11.7)	72%	BDI-II=31.8(10.7)	30.1(8.9)	1.27(1.15)	34%	CBT+TAU vs TAU	PHQ-9
GENPOD (2)	601	Adults 18–74 with depressive episode	38.8(12.4)	68%	BDI-II=33.7(9.7)	30.8(8.0)	1.68(1.37)	41%	Citalopram vs Reboxetine	BDI-II & HADS
IPCRESS (3)	295	Adults scoring ≥14 BDI-II and GP confirmed diagnosis of depression	34.9(11.6)	68%	BDI-II=33.2(8.8)	29.6(8.7)	1.44(1.25)	34%	iCBT+TAU vs TAU + waiting list for iCBT	BDI-II
MIR (4)	480	Adults ≥18 taking SSRIs or SNRIs at adequate dose for≥ 6 weeks, and scored ≥14 on BDI-II	50.7(13.2)	69%	BDI-II=31.1(9.9)	27.7(8.3)	1.04(1.04)	30%	Mirtazapine vs Placebo	BDI-II & PHQ-9
PANDA (5)	652	Adults presenting with low mood or depression to GP in last 2 years, free of ADM for 8 weeks up to baseline	39.7(15.0)	59%	BDI-II=23.9(10.3)	21.3(10.1)	1.22(1.19)	69%	Sertraline vs Placebo	BDI-II & PHQ-9
TREAD (6)	361	Adults 18–69 who met diagnostic criteria for MDD and scored ≥14 on BDI-II	39.8(12.6)	66%	BDI-II=32.1(9.2)	28.1(7.8)	1.49(1.28)	35%	Physical Activity + TAU vs TAU	BDI-II

Abbreviations: ADM – Antidepressant medication; BDI-II – Beck Depression Inventory; HADS – Hospital Anxiety and Depression Scale; iCBT (internet based therapist delivered cognitive behavioural therapy); MDD – Major Depressive Disorder; PHQ-9 – Patient Health Questionnaire – nine item version; T0 - Baseline; TAU – treatment as usual.

### 2.2 Measures

Three measures were used across all studies at baseline: the CIS-R which was an inclusion criterion (Lewis et al., 1992), used to determine durations of anxiety and depression, and diagnoses; the Beck Depression Inventory (BDI-II) (Beck et al., 1996); and although not specified as an inclusion criterion all eligible studies serendipitously used the same measure of life events, taken from the Adult Psychiatric Morbidity Surveys (McManus et al., 2016)() (based on the Social Readjustment Rating Scale) (Holmes and Rahe, 1967). On this scale participants indicate whether or not they experienced any major life events in the preceding six months, as such the measure allows for temporal considerations relevant to the secondary aim and some of the analytic models described in the data analysis section below. The following events are included in the measure: serious arguments/disputes; bereavement; problematic debt; divorce; serious illness/injury; being victim to a violent crime/assault; legal troubles; and being sacked/losing one's job. See Supplementary Materials for a full list of the questions from this scale. At 3–4 months, five studies used the BDI-II and one used the PHQ-9 (Kroenke et al., 2001), see Supplementary Table 3.

### 2.3 Ethical considerations, consent, and trial registrations

All studies were granted NHS Research ethical approvals and all participants gave informed consent (Supplementary Table 4). No additional ethical approval was required for this study: HRA reference 712/86/32/81.

### 2.4 Data handling and data management

#### 2.4.1 Data extraction

Data were extracted for each study participant on all variables in Table 2 by the chief investigators or data managers of each individual study and were cleaned one study at a time, independently by two reviewers (JB and RS), and cross-checked with publications and via liaison with chief investigators for each study. Issues were resolved by consensus between four reviewers (JB, RS, GL and SP). For further details see Supplementary Materials.

**Table 2:** Descriptive statistics of life events reported across the whole sample, and baseline characteristics of those reporting none compared to one or more life events in the six months pre-baseline.

**Self-reported Baseline Characteristics**	**Factor**	**Whole Sample N(%), or Mean(SD)**		
**Total Sample**	**N**	**2858**		
**Number of recent life events**	Mean(sd)	1.35(1.24)		
**Any life events**	No	817(28.59)		
	Yes	2041(71.41)		
**One event**		932(32.61)		
**Two events**		647(22.64)		
**Three or more events**		462(16.17)		
**Arguments**	Yes	674(23.59)		
**Bereavement**	Yes	539(18.87)		
**Debt**	Yes	958(33.54)		
**Divorce**	Yes	318(11.13)		
**Victim of violent crime/assault**	Yes	197(6.90)		
**Illness or Injury**	Yes	964(33.74)		
**Legal troubles**	Yes	233(8.16)		
**Sacked/Lost job**	Yes	178(6.23)		
		**No reported life events N(%), or Mean(SD)**	**One or more reported life events N(%), or Mean(SD)**	**χ2 or *t*-test p-value**
**Sample size**	**N**	817(28.6)	2041(71.4)	
**Age**	Mean(sd)	44.70(14.5)	41.64(13.9)	<0.0001
**Gender**	Female	541(66.2)	1359(66.7)	.82
	Male	276(33.8)	680(33.3)	
	Other	0	0	
**Ethnicity**	White	778(95.2)	1920(94.1)	.24
	Non-White	39(4.8)	120(5.9)	
**Employment status**	Employed	508(62.3)	1131(55.4)	<0.0001
	Not seeking employment	221(27.1)	464(22.7)	
	Unemployed	87(10.7)	445(21.8)	
**Marital Status**	Married/cohabiting	472(57.8)	907(44.4)	<0.0001
	Single	217(26.6)	694(34.0)	
	No longer married	128(15.7)	440(21.6)	
**Financial strain**	Doing OK	477(58.5)	707(34.7)	<0.0001
	Just about getting by	244(29.9)	670(32.9)	
	Struggling financially	95(11.6)	662(32.5)	
**Social Support Scale Score**	Mean(sd)	20.66(3.76)	20.08(3.89)	.0003
**Long-term physical health condition**	No	548(80.1)	1325(77.7)	.20
	Yes	136(19.9)	380(22.3)	
**Functional Impairment**	No	434(53.1)	914(44.8)	.0001
	Yes	383(46.9)	1127(55.2)	
**AUDIT-PC-Total Score**	Mean(sd)	2.51(2.63)	2.91(3.19)	.002
				
**First Life-time Depressive Episode**	No	624(76.4)	1624(79.6)	.0007
	Yes	193(23.6)	417(20.4)	
**History of Antidepressant treatment**	No	270(33.0)	638(31.3)	.35
	Yes	547(67.0)	1403(68.7)	
**Family history of depression**	No	316(40.6)	673(33.7)	.0007
	Yes	462(59.4)	1322(66.3)	
**CIS-R durations**	Depression	3.46(1.43)	3.41(1.34)	.35
	Average Anxiety Duration	2.05(1.02)	2.18(1.11)	.002
**Comorbid panic disorder**	No	764(93.5)	1859(91.1)	.033
	Yes	53(6.5)	182(8.9)	
**Baseline BDI-II score**	Mean(sd)	28.08(10.18)	31.38(10.52)	<0.0001

#### 2.4.2 Missing data

Missing data were imputed using multiple imputation with chained equations (MICE) in Stata 16.0, see Supplementary for further details.

### 2.5 Data analysis plan

Analyses for the main aim were conducted in line with the study protocol (Buckman et al., 2020), the subsidiary aim looking at two subgroups of patients reporting severe life events, for robustness, was not stated in the protocol. Details of other protocol amendments are noted in the Supplementary Materials. Associations between life events and prognosis were investigated controlling for treatment type and other factors as detailed below.

#### 2.5.1 Outcomes

The primary outcome was depressive symptoms at 3–4 months post-baseline, captured with: (1) the standardised and mean-centred score (z-score) on the primary depressive symptom measure (Table 1); and (2) the logarithm (“log outcome”) of those scores combined across studies. Exponentiating the regression coefficient provides an estimate of the percentage difference in symptoms at endpoint per unit change in the exposure variable. It was expected that these would yield similar results but that percentage differences might be more easily interpreted and do not require division by standard deviation estimates.

Secondary outcomes: 1) remission at 3–4 months (for definitions see Supplementary Table 3). 2) The z-score of the depressive symptom scale scores at 6–8 months.

#### 2.5.2 Prognostic factors

Prognostic associations were investigated for (1) the total number of reported life events, (2) any reported life event, (3) 1, 2, and 3 or more reported events (compared against no reported life events), and (4) each individual life event reported occurring within 6 months prior to entering treatment.

#### 2.5.3 Confounding

As causal pathways between potential confounding variables, life events, and prognosis are not known, we modelled associations with and without each potential confounder to evaluate the impact on reported associations. The randomization (treatments) in each study, age, ethnicity, and self-reported gender at baseline were adjusted for in all models. In order to adjust for treatment a single variable was created with dummy categories for each of the randomized groups in each of the studies. Markers of severity of depression previously found to be associated with prognosis independent of treatment (Buckman et al., 2021a) (depressive symptom severity, durations of depression and anxiety, history of antidepressant treatment, and comorbid panic disorder), were adjusted for in separate models. Social support, ethnicity, marital status, employment status and financial strain were additionally adjusted for as potential confounders in subsequent models (Buckman et al., 2021, 2021b, 2021a). Finally, only confounders that can be reasonably assumed to have been present prior to the life event (occurring up to six months pre-baseline) were adjusted for (this excludes clinical and demographic variables that are changeable through time and might have been affected by the life event; i.e. baseline depressive symptom severity, employment status, marital status, financial strain, and social support).

A number of other potential clinical confounding factors were considered but were not found to be independently associated with either the prognostic factors or the outcome variables so were not included in presented analyses. These were alcohol misuse, functional impairment, family history of depression, and long-term health condition status. See Supplementary Table 3 for details of how these were measured.

#### 2.5.4 Primary analyses

To consider the evidence for associations between each prognostic life event variable (see above) and outcomes four models were constructed:

1 Adjusted for the randomized treatment allocation in each study, age, ethnicity and gender.
2 As in 1 additionally adjusted for baseline BDI-II score; durations of depression and anxiety; history of antidepressant treatment; and comorbid panic disorder
3 As in 2 additionally adjusted for: social support; marital status; employment status; and financial strain.
4 As in 1 with the addition of any variables from Models 2 and 3 which must have occurred prior to the reported life events. For the primary aim this meant only a history of antidepressant treatment was added to the model, and for the subgroups assessed in the secondary aim other variables were included (as below). All other variables added in Models 2 and 3 which might have occurred after the reported life event(s) were removed.

For the primary aim, models were constructed with all participants, for the secondary aim, models were restricted to those with i) depressive episodes of at least six months duration at baseline (durations of depression and anxiety were therefore retained in Model 4); and ii) a first life-time episode of depression (so past antidepressant treatment was removed from all relevant models for this group so Model 4 was equivalent to Model 1).

Two-stage DerSimonian and Laird random effects meta-analyses were conducted with “admetan” in Stata 16. Heterogeneity was assessed with prediction intervals and the I^2^ statistic (Higgins et al., 2003). One-stage approaches have been favoured elsewhere (Cristea et al., 2019; Cuijpers et al., 2020; Karyotaki et al., 2017; Weitz et al., 2015). Such approaches are particularly useful when complex modelling techniques are required, but they can lead to increased bias in determining between-study effects (Fisher, 2015). As no complex modelling was necessary, the two-stage approach was considered most suitable (Fisher, 2015).

#### 2.5.5 Sensitivity analyses

Sensitivity analyses were conducted where heterogeneity was problematic (I^2^ above 75%) (Higgins et al., 2003), and where any studies were rated as having moderate or high risks of bias, or offered a low quality of evidence. Further analyses were conducted using the BDI-II score at 3–4 months excluding the one study that did not collect those data. For details and results of sensitivity analyses see Supplementary Materials.

### 2.6 Risk of bias and evidence quality

Two reviewers (JB & RS) independently rated the risk of bias in each study using the Quality in Prognosis Studies (QUIPS) tool (Hayden et al., 2013), and rated the quality of evidence for each prognostic indicator using the Grading Recommendations, Assessment, Development and Evaluations (GRADE) framework (Guyatt et al., 2008).

## 3 Results

### 3.1 Characteristics of the included studies

**Fig. 1:**
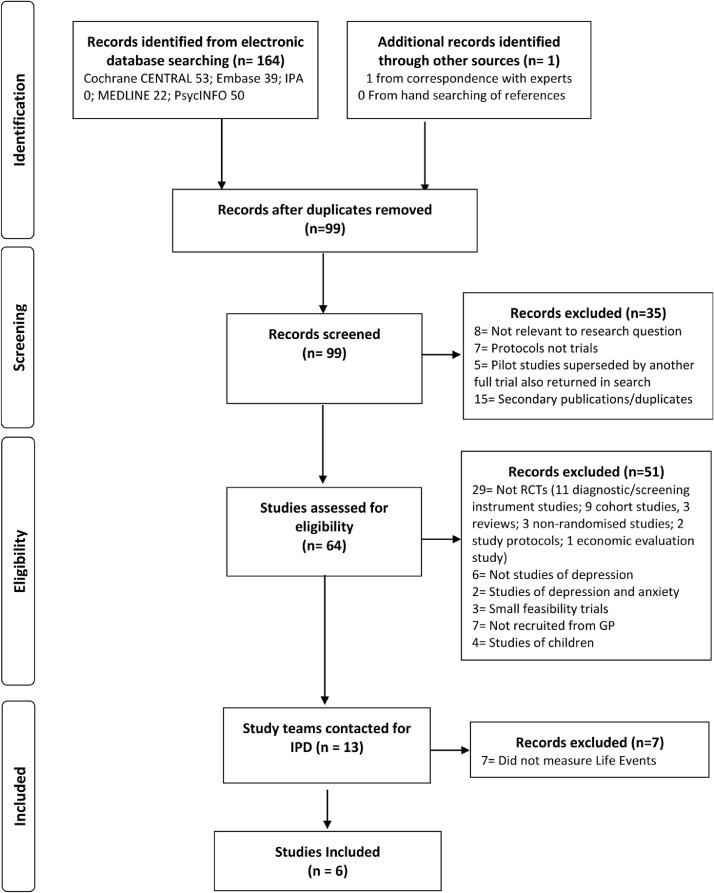
Flow of studies through selection process for IPD meta-analysis.

Six RCTs met inclusion criteria, all provided IPD (Fig. 1). Details of the studies are in Table 1. The studies included a number of commonly available treatments, including: antidepressant medications, cognitive behaviour therapy, and structured physical activity; and were tested against placebo, treatment as usual, or another antidepressant (Table 1).

### 3.2 Quality assessments and risk of bias

Two reviewers (JB and RS) independently judged the risk of bias in each study to be low in most domains, although one study was judged to have a moderate risk of bias due to attrition, and another was judged to have high risk of bias in this domain. Based on the GRADE framework, we considered the quality of evidence for life events as a prognostic indicator to be high (see Supplementary Table 5); interrater reliability was very high across both measures: Cohen's Kappa *k* = 0.98 for QUIPS and *k* = 1.00 for GRADE.

### 3.3 Baseline descriptive statistics

Most participants (71.4%) reported at least one life event within the six months prior to their baseline assessment: mean (SD) =1.35 (1.24); range 0–7. The most commonly reported events were suffering a serious illness/injury (33.7%) and problematic debt (33.4%). The least common events were losing one's job (6.2%) and being the victim of a violent crime (6.9%) (see Table 2). Compared to those reporting no life events, participants reporting at least one event were younger and were more likely to report: being unemployed; being single or no longer married; struggling financially; problems with social support; more alcohol use; and having a family history of depression. In addition, patients reporting any life events had significantly higher levels of severity in terms of baseline depressive symptoms, duration of anxiety, comorbid panic disorder, and functional impairment (Table 2).

Approximately two thirds (66.83%) of the sample had episodes of depression lasting six months or longer at baseline (*n* = 1910), and just over one in five of all participants (21.3%) was seeking treatment for a first life-time depressive episode (*n* = 610).

### 3.4 Prognosis for symptoms at 3–4 months

The total number of life events reported, and the reporting of any life event, were associated with prognosis independent of treatment type. For each additional life event, participants had higher depressive symptom scale scores (Tables 3, 4). This held both across the whole sample, and in the two subgroups of patients examined: those with at least six months duration of depression (Supplementary Tables 6–7), and those with a first life-time depressive episode (Supplementary Tables 8–9). When adjusting for markers of depressive severity (Model 2), the evidence for effects was weaker, and when additionally adjusting for baseline variables that may be more recent markers of the effect of the life event (Model 3), there no longer was evidence for associations between either the number of events reported or reporting any life events and prognosis at 3–4 months. When removing variables that might have been affected by the events or that occurred after the reported events (Model 4), there was evidence of associations with prognosis at 3–4 months (Tables 3, 4, Supplementary Tables 6–9; see Supplementary Figures 1–6 for between study heterogeneity). Patients reporting three or more major life events in the six months prior to baseline had considerably worse prognoses at 3–4 months, on average their depressive symptom scale scores were approximately 30% (95%CI: 18–33%) higher than those reporting no major life events in that time period (Table 4).

**Table 3:** Differences in mean depressive symptoms at 3–4 months post-baseline per unit increase in life event variables, across the whole sample (*N* = 2858).

**Life Events Variable**	**Adjusted for treatment, age, and gender**^		Additionally adjusted for depressive severity factors*		**Additionally adjusted for demographics and social support**‡		**Removing factors temporally after the reported life events**†	
	**Mean difference (95%CI)**	**I^2^**	**Mean difference (95%CI)**	**I^2^**	**Mean difference (95%CI)**	**I^2^**	**Mean difference (95%CI)**	**I^2^**
Life events total score	0.11(0.08 to 0.15)	23	0.05(0.00 to 0.09)	48	0.02(−0.02 to 0.05)	0	0.11(0.07 to 0.15)	32
Any life events	0.23(0.15 to 0.31)	0	0.12(0.01 to 0.22)	46	0.06(−0.02 to 0.14)	13	0.22(0.14 to 0.30)	0
Zero Life events (reference)								
One Life event	0.14(0.05 to 0.24)	0	0.09(−0.01 to 0.20)	30	0.07(−0.02 to 0.17)	21	0.14(0.05 to 0.23)	0
Two Life events	0.23(0.13 to 0.34)	0	0.11(−0.02 to 0.24)	36	0.04(−0.07 to 0.14)	0	0.22(0.11 to 0.32)	0
Three or More Life events	0.40(0.27 to 0.53)	0	0.20(0.04 to 0.36)	38	0.08(−0.05 to 0.22)	0	0.38(0.25 to 0.51)	0
Arguments	0.23(0.13 to 0.32)	0	0.08(−0.01 to 0.26)	0	0.05(−0.04 to 0.14)	0	0.21(0.12 to 0.31)	0
Bereavement	0.01(−0.09 to 0.11)	0	−0.01(−0.10 to 0.07)	0	−0.04(−0.12 to 0.05)	0	0.00(−0.09 to 0.10)	0
Debt	0.29(0.17 to 0.41)	16	0.15(0.02 to 0.28)	60	0.09(−0.04 to 0.22)	48	0.28(0.16 to 0.40)	44
Divorce	0.19(0.06 to 0.32)	0	0.12(−0.01 to 0.26)	19	0.05(−0.11 to 0.21)	33	0.19(0.06 to 0.32)	0
Victim of violent crime	0.29(0.12 to 0.46)	7	0.17(0.02 to 0.33)	12	0.11(−0.04 to 0.25)	0	0.27(0.11 to 0.43)	3
Illness or Injury	0.00(−0.16 to 0.15)	36	0.00(−0.10 to 0.11)	38	0.00(−0.10 to 0.11)	39	0.00(−0.15 to 0.15)	65
Legal troubles	0.16(0.01 to 0.30)	3	0.02(−0.15 to 0.20)	41	−0.03(−0.19 to 0.13)	31	0.14(−0.01 to 0.29)	7
Sacked/Lost job	−0.06(−0.22 to 0.09)	1	−0.06(−0.20 to 0.08)	0	−0.18(−0.35 to −0.01)	17	−0.08(−0.24 to 0.07)	0

^ adjusted for allocated treatment, gender, and age;.

⁎ adjusted for treatment, gender, age, ethnicity, baseline BDI-II score, average anxiety duration, depression duration, comorbid panic disorder, and history of antidepressant treatment;.

‡ adjusted for treatment, gender, age, ethnicity, baseline BDI-II score, average anxiety duration, depression duration, comorbid panic disorder, history of antidepressant treatment, social support, marital status, employment status, and financial strain;.

† adjusted for treatment, gender, age, ethnicity, and history of antidepressant treatment.

**Table 4:** Percentage differences in depressive symptoms at 3–4 months post-baseline per unit increase in life events variables, across the whole sample (*N* = 2858).

**Life Events Variable**	**Adjusted for treatment, age, and gender**^		Additionally adjusted for depressive severity factors*		**Additionally adjusted for demographics and social support**‡		**Removing factors temporally after the reported life events**†	
	**%(95%CI)**	**I^2^**	**%(95%CI)**	**I^2^**	**%(95%CI)**	**I^2^**	**%(95%CI)**	**I^2^**
Life events total score	7.86(4.68 to 11.14)	27	3.43(0.05 to 6.92)	43	1.41(−1.28 to 4.17)	7	7.54(4.37 to 10.80)	27
Any life events	15.96(8.64 to 23.78)	0	7.28(−2.00 to 17.45)	48	3.78(−4.03 to 12.23)	26	15.24(7.96 to 23.02)	0
Zero Life events (reference)								
One Life event	9.27(1.34 to 17.83)	0	5.08(−3.77 to 14.73)	27	4.51(−3.95 to 13.72)	18	8.97(1.03 to 17.54)	0
Two Life events	17.28(7.42 to 28.05)	6	6.87(−4.51 to 19.60)	41	3.05(−6.70 to 13.82)	20	16.49(7.00 to 26.83)	0
Three or More Life events	30.98(18.83 to 44.38)	3	14.71(1.93 to 29.10)	34	5.69(−4.98 to 17.55)	0	30.25(18.37 to 43.33)	0
Arguments	17.22(9.22 to 25.08)	0	6.37(−0.64 to 13.88)	0	3.31(−3.50 to 10.61)	0	16.50(8.54 to 25.04)	0
Bereavement	2.02(−5.13 to 9.72)	0	0.58(−6.39 to 8.08)	7	−0.65(−7.49 to 6.70)	6	1.91(−5.21 to 9.58)	0
Debt	21.94(9.21 to 36.15)	26	10.92(−0.22 to 23.29)	62	8.14(−2.46 to 19.90)	49	21.24(9.03 to 34.82)	59
Divorce	12.92(1.94 to 25.08)	0	8.86(−1.28 to 20.05)	0	2.30(−9.07 to 15.09)	24	13.17(2.17 to 25.35)	0
Victim of violent crime	25.78(12.22 to 40.98)	0	15.02(3.08 to 28.36)	7	−8.49(−2.56 to 20.78)	0	24.21(10.77 to 39.28)	0
Illness or Injury	−2.48(−11.53 to 7.49)	28	−1.62(−8.22 to 5.45)	13	−1.51(−8.49 to 6.01)	21	−2.10(−11.19 to 7.93)	48
Legal troubles	13.36(1.85 to 26.17)	0	4.60(−5.31 to 15.54)	0	0.32(−9.41 to 11.09)	0	12.32(0.91 to 25.02)	0
Sacked/Lost job	2.37(−9.76 to 16.14)	0	1.57(−9.89 to 14.49)	0	−6.83(−18.60 to 6.63)	14	1.04(−11.04 to 14.77)	0

^ adjusted for allocated treatment, gender, and age;.

⁎ adjusted for treatment, gender, age, ethnicity, baseline BDI-II score, average anxiety duration, depression duration, comorbid panic disorder, and history of antidepressant treatment;.

‡ adjusted for treatment, gender, age, ethnicity, baseline BDI-II score, average anxiety duration, depression duration, comorbid panic disorder, history of antidepressant treatment, social support, marital status, employment status, and financial strain;.

† adjusted for treatment, gender, age, ethnicity, and history of antidepressant treatment.

For most individual life events, there was evidence of associations with prognosis at 3–4 months (exceptions being bereavement, illness/injury, and losing one's job). This was evident both across the whole sample (Tables 3, 4) and within the two subsamples (Supplementary Tables 6–9). After adjusting for the markers of depressive severity (Model 2) again, evidence for all associations was weaker, however those reporting either problematic debt or being the victim of a violent crime had worse prognoses. After additionally adjusting for clinical and socio-demographic confounders (Model 3), there was no evidence of associations between any of the assessed individual life events and prognosis at 3–4 months in the whole sample. In the subsample of participants with durations of depression of at least six months at baseline there was some evidence that those who reported being the victim of violent crime had poorer prognoses at 3–4 months. In Model 4, when removing variables that could have occurred after the life events, there was good evidence for several types of events being associated with worse prognoses: reporting serious arguments/disputes, problematic debt, or being the victim of a violent crime, were associated with worse prognosis at 3–4 months (Tables 3, 4 & Supplementary Tables 6–7). On average, depressive symptom scale scores were between 17 and 24% higher for patients reporting such events compared to those not reporting them (Table 4). There was also some evidence that patients reporting legal troubles, or having recently gone through a divorce or separation, had poorer prognoses at 3–4 months, although with associations of lower magnitudes (Tables 3, 4). Among patients experiencing a first life-time depressive episode, generally there was a lack of associations between severe life events and prognosis at 3–4 months, except for patients reporting serious arguments or disputes, who had worse prognoses at 3–4 months (Supplementary Tables 8–9).

### 3.5 Prognosis for remission at 3–4 months

Similar to the primary outcomes analyses, significant patterns of association between the life events variables and remission at 3–4 months were found, except in Model 3 in which the only non-null association was between reporting three or more major life events and worse odds of remission (Supplementary Table 10). Fewer associations with remission and the individual life events were found in Model 2 compared to the primary outcomes, although in Model 4 after removing factors that might have occurred after the reported life events those who reported serious arguments/disputes, problematic debt, divorce, or being the victim of a violent crime all had worse odds of remission. Further, in the subsample with at least six months depressive episode duration, arguments/disputes were associated with lower odds of remission across all models (Supplementary Table 11). There was a lack of evidence for prognostic associations in the subsample that had a first life-time depressive episode after adjusting for the variables in Models 2, 3, or 4 (Supplementary Table 12).

### 3.6 Prognosis for symptoms at 6–8 months

There were similar patterns of association at 6–8 months as there were for prognosis at 3–4 months. However, when adjusting for all depressive severity factors in Model 2, losing one's job was associated with worse prognosis, and adjusting for all variables in Model 3 there was evidence of associations between reporting one event and worse prognosis at 6–8 months in the whole sample and in the subsample (Supplementary Tables 13–14). Again, there was a lack of evidence for any associations between the life events variables and prognosis at 6–8 months amongst those with a first life-time depressive episode (Supplementary Table 15).

### 3.7 Sensitivity analyses

For the primary aim, no sensitivity analyses were deemed necessary based on heterogeneity, risk of bias, or study quality. Findings were very similar to the primary analyses when using the BDI-II score at 3–4 months in the five studies that collected such data (Supplementary Tables 16–18). Heterogeneity was very high in the association between reporting being sacked/losing one's job and prognosis at 3–4 months for the subgroup of patients experiencing their first life-time depressive episode, removing the COBALT study (Wiles et al., 2013) from these models resulted in no substantive changes in the results (Supplementary Table 19).

## 4 Discussion

There was evidence of associations between reporting major life events in the six months prior to seeking treatment for depression and prognosis independent of treatment type. Overall, patients who reported any severe life event had worse prognoses compared to patients who reported no such events. The strength of the association increased when more events were reported in an approximately monotonic fashion; those reporting three or more events had considerably worse prognoses than patients reporting no life events. This was true irrespective of whether or not the depressive episode started prior to the life events being experienced, but there was a lack of evidence for such an effect amongst those that had a first life-time depressive episode. The evidence for associations with prognosis across the whole sample for some types of events was stronger than for others. Reporting serious arguments/disputes, problematic debt, divorce, being the victim of a violent crime, or losing one's job were all associated with worse prognosis.

Adjusting for variables that might routinely be assessed in clinical practice (such as baseline depressive symptom severity) (Buckman et al., 2021a) attenuated these associations, and further adjusting for baseline variables that might have occurred after the life event or may have been affected by the life event (employment status, marital status, financial strain, and social support) resulted in few associations with prognosis, although there were notable exceptions. We do not know if these factors are confounders or whether they might lie on the causal pathway between life events and prognosis. However, our analysis of the subgroup with episodes of depression that preceded the reported life events yielded very similar results, supporting the notion that these were confounders. It is not known whether such episodes were preceded by any severe life events beyond those reported by participants of the included studies though, given the six month time period set out in the life events measure used in all of the included studies. So, we cannot determine whether the life events may themselves lie on the causal pathway between the above social and demographic factors and prognosis. Another question that arises from this is whether or not there are third factors or residual confounders that both give rise to the life events and eventuate in worse prognoses. Early life stress and chronic stressors are associated with greater likelihood of experiencing major life events, with experiencing depression (more often thought to lead to vulnerability to depression), and with worse prognoses for adults with depression (Buckman et al., 2018; Gourion, 2009; Kendler and Gardner, 2016; Monroe et al., 2007; Nanni et al., 2012). We had no data on these different types of stressors, but were particularly concerned with the prognoses of patients reporting a major life event recent to seeking treatment. Such events are likely to be reported more commonly in clinics in the comings months and years during and following the COVID-19 pandemic (Holmes et al., 2020), although it is also likely that the pandemic is producing chronic stress too (Öngür et al., 2020). Mediation effects or causal relationships could not be determined here, so this is a question for further research, but in any case, it is reasonable to conclude that current employment, marital status, financial strain, and social support may be better indicators of prognosis than life events.

### 4.1 Limitations

This was the first study to investigate the associations between life events and prognosis independent of a range of commonly available treatments for depression. The findings were based on a large IPD dataset, including complete data from all eligible studies, minimising selection bias. However, as only a little over one fifth of the sample was experiencing a first life-time episode, there may not have been sufficient power to detect effects in this subgroup.

All studies used the same measures of baseline characteristics, minimising bias in harmonising the data (Lesko et al., 2018). Data were extracted, cleaned, and checked by multiple reviewers, adding robustness to the methods (Buscemi et al., 2006), and all studies recruited participants in a primary care setting, so the findings here may be generalizable to a large proportion of depressed patients (McManus et al., 2016) . Some of the findings may have been affected by other selection biases; for example, it is unlikely that participants in RCTs are representative of all depressed patients. However, all but one of the RCTs included here were pragmatic trials, reducing selection biases and potentially improving generalizability (Rothwell, 2005).

All studies meeting our eligibility criteria used the same scale for assessing major life events, again reducing bias that might have been introduced harmonising across different scales (Lesko et al., 2018). However, the validity of life events scales has often been criticised for being unreliable with both under reporting and over reporting of events, and a lack of ability to reliably distinguish between chronic and acute events (Harkness and Monroe, 2016). It is possible that by only including studies set in primary care, for those meeting inclusion criteria the use of such measures was necessary for pragmatic reasons, given the typically very brief length of consultations in this setting. It is noteworthy that the originators of the abbreviated scale used here mitigated some common problems by removing non-stressful events and specifying a six-month time period, reducing some recall biases (McManus et al., 2016). That notwithstanding, the six month time period may be too long for accurate recall and to assess the impact of the events as acute stressors (Monroe et al., 2019). As there was no question on specifically when the event(s) occurred this may have introduced additional bias into our subgroup analyses of those whose depressive episodes predated the life event(s). Further, some of the questions on the scale require subjective interpretations that may lead to measurement error, for example those questions that qualify the degree of severity of the event such as “serious arguments or disputes with a close friend/relative or neighbour” give rise to ‘intracategory variation’, and are particularly prone to bias when respondents have depression (Dohrenwend, 2006). We presented results for all individual life events, so prognostic associations for items that are less prone to ‘intracategory variation’ (e.g. bereavement, divorce, and being sacked/losing one's job) were also demonstrated. In addition, we sought to mitigate problems due to reverse causality, investigating associations with prognosis separately in a group of patients with chronic depression whose depressive episodes preceded the assessed events. There are other potential sources of recall bias as well. The self-reported nature of the events may have given rise to further problems: depressed patients often exhibit cognitive biases which effect recall of negative events (Roiser et al., 2012), such biases are associated with treatment outcomes (Buckman et al., 2019), possibly confounding the associations between life events and prognosis. Adjustments were made for a number of confounders, but as noted above, residual confounding cannot be ruled out.

The use of a standardized outcome has been criticized, but the results using the z-score outcome were similar to those with the log outcome and the secondary and sensitivity outcomes, suggesting no substantive impact on the results. Alternative outcomes may be of use in future research. For example, if studies incorporate more regular outcome measurement schedules, they could provide a more fine-grained assessment over time of the impact of major life events on outcomes. Indeed, it would be informative for future research to address the hypothesis that severely stressful acute events lead to a longer time to remission, even if they do not greatly impact overall prognosis 3–4 months after commencing treatment.

### 4.2 Implications and conclusions

Stressful life events are common; in the present sample, over 70% reported at least one event in the past 6 months. The COVID-19 pandemic and governmental responses to it have resulted in increases in people experiencing major life events, particularly serious illness (WHO, 2020; Xiao and Torok, 2020), bereavement (Gunnell et al., 2020), unemployment and problematic debt (Gangopadhyaya and Garrett, 2020; Gunnell et al., 2020; Holmes et al., 2020; Xiao and Torok, 2020). We would expect that on average, people experiencing such events will be more likely to feel distressed as a result of the events and will be at greater risk of becoming depressed, whether that be with a first life-time episode or a recurrent one (Kendler and Gardner, 2016; Monroe et al., 2019).

This study has shown that life events may play a role in prognosis for patients seeking treatment for depression in primary care, regardless of treatment type, and whether or not they had chronic depression or were presenting with a first life-time depressive episode. However, the effects were largely shared with variables that might have been affected by the events (depressive severity, social support, marital status, employment status, and financial strain) and there are a number of important problems with the use of solely self-report checklist measures of life events. So, it may be most informative for prognosis to first assess for clinical, socio-demographic, and contextual prognostic factors and when considering life events to use a more thorough method of assessment to consider the ongoing impact of any life events at the point the patient presents. This does not mean that clinicians should not ask their patients about experiences of any such life events; indeed, it may be helpful to routinely ask patients whether they have experienced any major life events recently, and use information about any reported events to consider treatment options. The results of this study support further investigation of onward referrals for additional support specific to particular types of events (e.g. for debt advice, arbitration or mediation services for those with marital problems or serious disputes, victim support organisations, or employment advisors) to mitigate poorer prognosis (Fournier et al., 2009; Maslow, 1943; Van Der Lem et al., 2013).

## Statements

### Data availability

Requests for sharing of the IPD used in this study can be made to the corresponding author, any sharing of data will be subject to obtaining appropriate agreements from the chief investigators or data custodians for each individual trial dataset used here.

### Role of the funding source

This research was funded by the Wellcome Trust [20129/Z/16/Z], the MQ Foundation (for ZC: MQDS16/72), the Higher Education Funding Council for England (RS, PB, l-LA, IO, C'OD, and SP), the National Institute of Health Research (NIHR), NIHR University College London Hospitals Biomedical Research Centre (RS, PB, l-LA, IO, and SP), University College London (GA, GL), University College London (SDH), University of Southampton (TK), University of Exeter (EW), and University of York (SG). NIHR Biomedical Research Centre at the University Hospitals Bristol and Weston NHS Foundation Trust and the University of Bristol (NW: The views expressed are those of the authors and not necessarily those of the NIHR or the Department of Health and Social Care). Alzheimer's Society (grant code: 457 (AS-PG-18–013) for JS). National Institute for Health Research (NIHR) Biomedical Research Centre at South London and Maudsley NHS Foundation Trust and King's College London (TE and MS: The views expressed are those of the author(s) and not necessarily those of the NHS, the NIHR or the Department of Health).

The studies that individual patient data for this study were funded by:

1 COBALT: This research was funded by the National Institute for Health Research Health Technology Assessment (NIHR HTA) programme (project number 06/404/02).
2 GENPOD: Medical Research Council and supported by the Mental Health Research Network.
3 IPCRESS: BUPA Foundation
4 MIR: National Institute for Health Research (NIHR) Health Technology Assessment (HTA) programme (project 11/129/76) and supported by the NIHR Biomedical Research Centre at University Hospitals Bristol and Weston NHS Foundation Trust and the University of Bristol
5 PANDA: NIHR Programme Grant for Applied Research
6 TREAD: National Institute for Health Research (NIHR) Health Technology Assessment (HTA) programme.

The funding sources had no role in the design and conduct of the study; collection, management, analysis, and interpretation of the data; preparation, review, or approval of the manuscript; and decision to submit the manuscript for publication.

## Supplementary Materials

Supplementary Table 1–19

Supplementary Figure 1–6

## CRediT authorship contribution statement

**Joshua E.J. Buckman:** Conceptualization, Funding acquisition, Writing – original draft, Formal analysis, Writing – review & editing, Data curation. **Rob Saunders:** Writing – original draft, Formal analysis, Writing – review & editing, Data curation. **Laura-Louise Arundell:** Formal analysis, Writing – review & editing, Data curation, Writing – original draft. **Iyinoluwa D. Oshinowo:** Formal analysis, Writing – review & editing, Data curation, Writing – original draft. **Zachary D. Cohen:** Writing – original draft, Writing – review & editing, Formal analysis, Data curation. **Ciaran O'Driscoll:** Formal analysis, Writing – review & editing, Data curation, Writing – original draft. **Phoebe Barnett:** Formal analysis, Writing – review & editing, Data curation, Writing – original draft. **Joshua Stott:** Formal analysis, Writing – review & editing, Data curation, Writing – original draft. **Gareth Ambler:** Funding acquisition, Writing – original draft, Formal analysis. **Simon Gilbody:** Funding acquisition, Writing – original draft, Formal analysis, Writing – review & editing. **Steven D. Hollon:** Funding acquisition, Writing – original draft, Formal analysis. **Tony Kendrick:** Funding acquisition, Writing – original draft, Formal analysis, Writing – review & editing. **Edward Watkins:** Funding acquisition, Writing – original draft, Formal analysis, Writing – review & editing. **Thalia C. Eley:** Formal analysis, Writing – review & editing, Data curation, Writing – original draft. **Megan Skelton:** Formal analysis, Writing – review & editing, Data curation, Writing – original draft. **Nicola Wiles:** Writing – review & editing, Data curation, Writing – original draft. **David Kessler:** Writing – review & editing, Data curation, Writing – original draft. **Robert J. DeRubeis:** Funding acquisition, Writing – original draft, Formal analysis, Writing – review & editing. **Glyn Lewis:** Conceptualization, Funding acquisition, Writing – original draft, Data curation, Writing – review & editing, Formal analysis. **Stephen Pilling:** Conceptualization, Funding acquisition, Writing – original draft, Formal analysis, Data curation, Writing – review & editing.

## Declaration of Competing Interest

None.

## Appendix Supplementary materials

Image, application 1

Image, application 2
