# Supplementary material for: Life events and treatment prognosis for depression: A systematic review and individual patient data meta-analysis
Source: J Affect Disord. 2022 Feb 15;299:298–308. doi: 10.1016/j.jad.2021.12.030 (PMC9113943; doi:10.1016/j.jad.2021.12.030)
Supplement: Supplementary file 1 [file mmc1.docx]

# Supplementary Materials

## Details of review of systematic reviews and meta-analyses

Searches were run on the Cochrane database of systematic reviews, the Prospero register of systematic reviews, Embase, and Medline. Details of the search terms and results from the searches can be found in Supplementary Table 1. Across the databases 632 articles remained after removing duplicates, 71 of these were somewhat relevant and were read in full, from which 29 were directly relevant as they identified patient characteristics associated with prognosis for adults with depression. Only two of these 29 studies investigated the association between life events and prognosis.

**Supplementary Table 1.** Bibliographic database searches and results for literature review on indicators of prognosis for adults with depression

| **Searches** | **Results** |
| --- | --- |
| **Cochrane Database of Systematic Reviews (last searched on 27^th^ February 2020)** |  |
| 1. (depression or MDD or Major Depression or depressive episode): ti.ab.kw (Word variations have been searched) | 656 |
| 2. AND (Prognosis or Outcome): ti.ab.kw (Word variations have been searched) | 574 |
| 3. AND (systematic review or meta-analysis or meta analysis): ti,ab,kw (Word variations have been searched) | 350 |
| 4. NOT (psychosis or bipolar or bi-polar) | 330 |
| 5. Limit to Topic “Mental Health” | **131** |
| **Prospero (last searched on 27^th^ February 2020)** |  |
| 1. (depression or Depressive disorder or Major depression or Unipolar depression or MDD) | 5785 |
| 2. Filter in Health area of review “Mental health and behavioural conditions, or Public health (including social determinants of health)” | 2302 |
| 3. Filter in Type and method of review “Epidemiologic, Prognostic, Systematic Review, Meta-analysis, Individual patient data (IPD) Meta-analysis, Network meta-analysis, Review of reviews, or Qualitative synthesis” | 2254 |
| 4. Filter in Status of Review “Published” | **136** |
| **Embase searched 1974 to 2020 February 28** |  |
| 1. (Major depression or MDD or Major Depressive Disorder).m_titl. | 18745 |
| 1. (minor depression or MinD).m_titl | 13201 |
| 1. (depressive or depressive episode or depressive disorder).m.titl | 39836 |
| 1. Depression.m_titl. | 125022 |
| 1. 1 or 2 or 3 or 4 | 176324 |
| 1. treatment outcome/ | 839098 |
| 1. treatment response.mp. or treatment response/ | 271731 |
| 1. prognosis.mp. or prognostic.m[p. or prognostic assessment/ | 4939326 |
| 1. moderator | 8831 |
| 1. systematic review.mp. or “systematic review”/ or meta analysis/ | 385120 |
| 1. 2 or 3 or 4 or 5 | 1477081 |
| 1. 1 and 6 and 7 | 1251 |
| 1. (children or adolescent or child).mp. [mp=title, abstract, heading word, drug trade name, original title, device manufacturer, drug manufacturer, device trade name, keyword, floating subheading word, candidate term word] | 3167378 |
| 1. 12 NOT 13 | 1149 |
| 1. (old age or geriatric).mp. [mp=title, abstract, heading word, drug trade name, original title, device manufacturer, drug manufacturer, device trade name, keyword, floating subheading word, candidate term word] | 142854 |
| 1. 14 NOT 15 | 1106 |
| 1. bipolar disorder/ or bipolar depression/ or bipolar.mp. or psychosis.mp. or psychotic.mp. or schizoaffective.mp. or schizophrenia.mp. [mp=title, abstract, heading word, drug trade name, original title, device manufacturer, drug manufacturer, device trade name, keyword, floating subheading word, candidate term word | 355747 |
| 1. 16 NOT 17 | 962 |
| 1. (stroke or dementia or parkinson* disease or brain injury).mp. [mp=title, abstract, heading word, drug trade name, original title, device manufacturer, drug manufacturer, device trade name, keyword, floating subheading word, candidate term word] | 893303 |
| 1. 18 NOT 19 | 900 |
| 1. Limit 20 to (human and English and journal) | **864** |
| **Ovid MEDLINE 1946 to March Week 3 2019 (last searched on 27^th^ February 2020)** |  |
| 1. exp major depression/ or exp "depression (emotion)"/ or exp Depressive Disorder/ | 115136 |
| 2. prognosis.mp. | 726849 |
| 3. exp Treatment outcome/ | 1028415 |
| 4. 2 or 3 | 1656920 |
| 5. systematic review.mp. or “Systematic Review”/ | 169051 |
| 6. meta-analysis.mp. or Meta-Analysis/ | 179131 |
| 7. 5 or 6 | 271962 |
| 8. 1 and 4 and 7 | 578 |
| 9. limit 8 to (English language and humans and "reviews (maximizes specificity)") | **513** |

## Details of search terms and search results for RCTs to form IPD dataset

**Supplementary Table 2.** Bibliographic database searches and results

| **Searches** | **Results** |
| --- | --- |
| **Cochrane CENTRAL Trial Register (searched on 8^th^ October 2021)** |  |
| 1. ("Depression" or "MDD" or "Unipolar" or "Depressive"):ti,ab,kw (Word variations have been searched) | 87938 |
| 2. (“RCT” or "controlled trial" or "randomized controlled trial" or "clinical trial"):ti,ab,kw (Word variations have been searched) | 1144748 |
| 3. ("CIS-R" or "Clinical Interview Schedule" or “Revised Clinical Interview Schedule” or “Clinical Interview Schedule Revised”):ti,ab,kw (Word variations have been searched) | 67 |
| 4. #1 and #2 and #3 | **53** |
| **Embase 1947 to 2021 October 07** |  |
| 1. (depression or Depressive disorder or Major depression or Unipolar depression or MDD).mp. | 769534 |
| 2. exp controlled clinical trial/ or exp "randomized controlled trial (topic)"/ or exp "clinical trial"/ | 1859661 |
| 3. ("Clinical Interview Schedule" or "CIS-R" or "CISR" or "Revised clinical interview schedule" or "clinical interview schedule revised").af. | 893 |
| 4. 1 and 2 and 3 | **39** |
| **International Pharmaceutical Abstracts 1970 to September 2021** |  |
| 1. (depression or Depressive disorder or Major depression or Unipolar depression or MDD).mp. | 10859 |
| 2. (RCT or controlled trial or randomized controlled trial or clinical trial).mp. | 16472 |
| 3. ("Clinical Interview Schedule" or "CIS-R" or "CISR" or "Revised clinical interview schedule" or "clinical interview schedule revised").af. | 4 |
| 4. 1 and 2 and 3 | **0** |
| **Ovid MEDLINE 1946 to October 07, 2021** |  |
| 1. exp major depression/ or exp "depression (emotion)"/ | 132832 |
| 2. exp Depressive Disorder, Major/ | 33340 |
| 3. exp Depressive Disorder, Major/ or exp Depressive Disorder/ or exp Depression/ | 233903 |
| 4. 1 or 2 or 3 | 233903 |
| 5. exp controlled clinical trial/ or exp "randomized controlled trial (topic)"/ | 636694 |
| 6. ("Clinical Interview Schedule" or "CIS-R" or "CISR" or "Revised clinical interview schedule" or "clinical interview schedule revised").af. | 651 |
| 7. 4 and 5 and 6 | **22** |
| **PsycINFO 1806 to October Week 1 2021** |  |
| 1. exp major depression/ or exp "depression (emotion)"/ | 166578 |
| 2. (depression or Depressive disorder or Major depression or Unipolar depression or MDD).mp. | 361622 |
| 3. 1 or 2 | 361850 |
| 4. exp "randomized controlled trial (topic)"/ or exp "clinical trial"/ or exp "controlled trial"/ or exp "randomized clinical trial"/ | 12990 |
| 5. (RCT or controlled trial or randomized controlled trial or clinical trial).mp. | 47329 |
| 6. 4 or 5 | 54073 |
| 7. ("Clinical Interview Schedule" or "CIS-R" or "CISR" or "Revised clinical interview schedule" or "clinical interview schedule revised").af. | 1275 |
| 8. 3 and 6 and 7 | **50** |

No limits or filters were set on searches.

**Supplementary Table 3.** Measures used across the studies of the Dep-GP IPD database of relevance to this study

| **Measure** | **Details** | **Scores and Cut-offs** |
| --- | --- | --- |
| The CIS-R (Lewis et al., 1992) | Consists of 14 symptom subsections scored 0-4 covering core features of depression, depressive thoughts (scored 0-5), fatigue, concentration/forgetfulness, and sleep, generalized anxiety, worry, irritability, obsessions, compulsions, health anxiety, somatic concerns, phobic anxiety (split into agoraphobia, social phobia, and specific phobia), and panic. A final section measures general health, impairment and weight change. | The total score ranges from 0-57 with a cut-off of ≥12 used to indicate likely common mental disorder, primary and secondary diagnoses using ICD-10 criteria are given, as are binary indictors of diagnosis for all the disorders assessed. Scores of <12 among those that were previously depressed can be used to indicate remission. |
| Beck Depression Inventory 2^nd^ Edition (BDI-II) (Beck et al., 1996) | Consists of 21 items to assess depressive symptoms, each item is scored 0-3. | There is a maximum score obtainable of 63, and a cut-off of ≥10 is used indicate significant symptoms of depression, scores of <10 are therefore used to indicate remission in those that were previously depressed/scored ≥10. |
| Patient Health Questionnaire 9-item version (PHQ-9) (Kroenke et al., 2001) | This is a depression screening measure, with respondents asked to rate how often they have been bothered by each of the nine symptom items over the preceding two weeks. Each item is scored 0-3 | There is a maximum score of 27 with a cut-off of ≥10 is used to indicate “caseness” for depression, a score of 9 or below for those that were previously depressed is therefore considered to indicate remission |
| Life Events Scale: taken from the Adult Psychiatric Morbidity Survey (McManus et al., 2016) based upon the Social Readjustment Rating Scale (Holmes and Rahe, 1967) | Participants are asked to respond yes/no to whether they have suffered any of eight events within the last six months: 1) serious arguments or disputes; 2) a bereavement; 3) problematic debt that could not be paid back if it had to be (excluding mortgages); 4) divorce/separation; 5) serious illness or injury; 6) being victim to a violent crime or assault; 7) being sacked or losing one’s job; and 8) having problems with the police or other legal troubles. Each item is scored yes (1) or no (0) and the total score is the sum of all the items. Questions for this scale are listed below* | N/A |
| All measures apart from the PHQ-9 were used in all six studies, PHQ-9 was used in three studies (COBALT, MIR, & PANDA), | | |

*Questions from the Life Events Scale:

The next few questions are about events that may have happened to you in the PAST SIX MONTHS:

1. Has someone close to you died in the PAST SIX MONTHS?
2. Have you separated/divorced from your spouse/partner in the PAST SIX MONTHS?
3. In the PAST SIX MONTHS has a serious illness or injury occurred to yourself or someone close to you?
4. In the PAST SIX MONTHS has a mugging, burglary or other serious assault happened to you or someone close to you?
5. In the PAST SIX MONTHS have you or someone close to you had problems with the Police involving a court appearance?
6. Do you have any outstanding debts that you could not pay if you had to? (DO NOT include the total value of your mortgage, but DO include overdrafts and any rent or mortgage ARREARS).
7. In the PAST SIX MONTHS have you had a serious dispute with a close friend/relative or neighbour?
8. In the PAST SIX MONTHS have you been made redundant or sacked from your job?"

## Ethical Approvals and Trial Registrations details for studies included in Dep-GP IPD dataset

**Supplementary Table 4.** Ethical approval and Trial Registration details of the studies included in the Dep-GP IPD database

| **Study** | **Ethical Approvals** | **Trial Registration details** |
| --- | --- | --- |
| COBALT | Approvals were granted by West Midlands Research Ethics Committee (NRES/07/H1208/60) and research governance approval was obtained from the local Primary Care Trusts/Health Boards | ISRCTN38231611; https://doi.org/10.1186/ISRCTN38231611 |
| GENPOD | Approvals granted by South West Research Ethics Committee (MREC 02/6/076) and research governance approval was granted by Bristol, Manchester and Newcastle Primary Care NHS Trusts. | ISRCTN31345163; https://doi.org/10.1186/ISRCTN31345163 |
| IPCRESS | Approval granted by Royal Free and Hampstead Research Ethics Committee, reference number 05/Q0501/18 | ISRCTN45444578; https://doi.org/10.1186/ISRCTN45444578 |
| MIR | Approvals were granted by South East Wales Research Ethics Committee Panel C (ref: 12/WA/0353); Bristol Clinical Commissioning Group (CCG), and other CCGs provided research governance assurance. | ISRCTN06653773; https://doi.org/10.1186/ISRCTN06653773 |
| PANDA | Ethical approval was granted by Bristol Research Ethics Committee Centre (12/SW/0267). | ISRCTN84544741; https://doi.org/10.1186/ISRCTN84544741 |
| TREAD | Approvals were granted by West Midlands multicentre research ethics committee (MREC 05/MRE07/42), and research governance approval was given by the relevant local National Health Service primary care trusts | ISRCTN16900744; https://doi.org/10.1186/ISRCTN16900744 |

## Additional Details of Methods and Data Analyses

## Quality assessments and Risk of Bias

**Supplementary Table 5.** Risk of Bias and Quality Ratings for each of the six included studies.

|  |  |  | | | | | |
| --- | --- | --- | --- | --- | --- | --- | --- |
| **QUIPS Risk of Bias Ratings** | **Study** | **Study Participation** | **Study Attrition** | **Prognostic Factor Measurement** | **Outcome Measurement** | **Study Confounding** | **Statistical Analysis and Reporting** |
|  | COBALT | Low | Low | Low | Moderate | Low | Low |
|  | GENPOD | Low | Low | Low | Low | Low | Low |
|  | IPCRESS | Low | High | Low | Low | Low | Low |
|  | MIR | Low | Moderate | Low | Low | Low | Low |
|  | PANDA | Low | Low | Low | Low | Low | Low |
|  | TREAD | Low | Low | Low | Low | Low | Low |
|  | **Overall** | **Low** | **Low** | **Low** | **Low** | **Low** | **Low** |
| **GRADE Quality Assessment** | **Study** | **Risk of Bias** | **Imprecision** | **Inconsistency** | **Indirectness** | **Publication bias** | **Overall Quality per Study** |
|  | COBALT | ++++ | +++ | ++++ | N/A | N/A | **High** |
|  | GENPOD | ++++ | +++ | ++++ | N/A | N/A | **High** |
|  | IPCRESS | ++++ | +++ | ++++ | N/A | N/A | **High** |
|  | MIR | ++++ | +++ | ++++ | N/A | N/A | **High** |
|  | PANDA | ++++ | +++ | ++++ | N/A | N/A | **High** |
|  | TREAD | ++++ | +++ | ++++ | N/A | N/A | **High** |
|  | Overall rating per category across dataset | ++++ | ++++ | ++++ | ++++ | ++++ |  |
|  | **Overall GRADE Rating** | **High** | | | | | |

N/A = Not Applicable

### Data handling and data management

#### Data Integrity Checks

Integrity of all baseline and endpoint data for each study were checked with the study team and against details published about each study. The numbers of participants included in the individual patient dataset for some studies is very slightly different than those in the published articles about the individual studies. This is because a very small number of cases were removed from Dep-GP if they had missing data on over 75% of the variables at baseline or were missing all CIS-R variables. This resulted in two patients being removed from the “IPCRESS” study (Kessler et al., 2009) and one from the “PANDA” study (Lewis et al., 2019).

#### Missing Data

Missing data were imputed using multiple imputation with chained equations (MICE) in [Stata](https://www.stata.com/) 16.0. This approach uses regression models to impute missing values. A number of imputed datasets (here we used 50) are produced to reflect the uncertainty/variability in the imputation process. Data not reasonably able to be log transformed to meet normality assumptions, were imputed using predictive mean matching (PMM) via a k-nearest neighbours approach as it is considered to be more appropriate for non-normal continuous variables (Horton and Lipsitz, 2001), here we used k=10. Linear regression was used for approximately normally distributed continuous variables, logistic regression models for binary variables, and ordinal and multinomial regression models for ordered and unordered categorical variables respectively. All imputation models were built using data on baseline and outcome variables following conventions (Royston and White, 2011). Only variables with less than 50% missing data were imputed. All imputation models were run to produce 50 imputed datasets. If the primary analysis showed that results differed considerably when studies with systematically missing baseline data were included/excluded from the meta-analytic models, then a separate imputation approach would have been taken, to impute these systematically missing data using multiple imputation with multilevel random effects for study (Resche-Rigon et al., 2013)

#### Software & Packages

Stata SE 16 (StataCorp, 2019): ipdmetan (Fisher, 2015), mvmeta (White, 2011), MICE(Royston, 2009), mi impute pmm (Morris et al., 2014) packages.

### Protocol amendments

We registered the process of finding studies and the research questions for this study on PROSPERO (CRD42019129512) and produced a protocol paper which was amended twice (Buckman et al., 2020). Below we explain the amendments made, the process of finding studies and forming the dataset for this study.

We started this project with one of the senior investigators (GL) in possession of individual patient data from two studies for which he was the chief investigator, and a third study that he was in the process of conducting. We ran scoping searches, noted that the CIS-R was the most commonly used comprehensive measures of depressive and anxiety symptoms and disorders in RCTs of depression set in primary care, and refined our scoping searches to look for studies that used the CIS-R. That author (GL) was a co-investigator on a number of other trials that used the CIS-R and we made contact with the chief investigators of those studies to ask for in-principle agreement to access IPD from their trials and then applied for funding for this project. Once funding was in place, we registered our project on PROSPERO, at that point we had run two rounds of searches (scoping searches and one set to inform our funding application), and we had obtained IPD data from four studies. We refined our searches by including two other databases and reaching out to experts for missed studies, this helped us find further studies. We invited the chief investigators from each of those studies to join the project. We began to collect some further IPD from the studies that had agreed to take part. We then wrote up a protocol paper with information of what we would do with those IPD data once the dataset was complete. We ran further searches and found one more study just before initially submitting the protocol paper. It was a protracted process to gain IPD from that study but the idea was that the Dep-GP IPD dataset would be formed from all of those studies we had found. The protocol paper was peer-reviewed and we amended it post-review to give more details about this process. The protocol was then accepted for publication. It was amended once more when we decided that our choice of an I^2^ threshold for considering problematic heterogeneity was too high, we dropped it from 80% to 75% for all models and to 50% for the final models, in line with recommendations (Higgins et al., 2003). We ran the final searches for studies meeting our inclusion criteria a few months before submitting this manuscript for publication and found no new studies meeting our criteria.

Our protocol paper provides information about all data we sought to extract from the included studies and all outcomes of interest. For the present study we were particularly interested in life events and potential confounders of any association between life events and prognosis. We constructed some exploratory Directed Acyclic Graphs (Shrier and Platt, 2008) to help consider what those confounders might be and limited the data used for this study to those factors (and life events). For the present study, we amended our inclusion criteria slightly to exclude studies that did not include a measure of life events at the baseline assessment. There were two changes to the statistical analysis plan that should be noted: firstly, we did not include attrition as an outcome for the present study due to limited data on the reasons for attrition and amount of treatment received at the point of termination from each included study, and secondly we added the secondary aim to consider prognostic associations in two clinically important subgroups of patients.

## Supplementary Results

**Supplementary Table 6.** Differences in mean depressive symptoms at 3-4 months post-baseline per unit increase in life events variables, for participants with at least six months duration of depression at baseline (N=1910).

| **Life Events Variable** | **Adjusted for treatment, age, and gender^** | | **Additionally adjusted for depressive severity factors*** | | **Additionally adjusted for demographics and social support‡** | | **Removing factors temporally after the reported life events⸷** | |
| --- | --- | --- | --- | --- | --- | --- | --- | --- |
|  | **Mean difference (95%CI)** | **I^2^** | **Mean difference (95%CI)** | **I^2^** | **Mean difference (95%CI)** | **I^2^** | **Mean difference (95%CI)** | **I^2^** |
| Life events total score | 0.14(0.10 to 0.18) | 0 | 0.07(0.02 to 0.12) | 39 | 0.03(-0.02 to 0.08) | 30 | 0.10(0.05 to 0.14) | 23 |
| Any life events | 0.24(0.14 to 0.34) | 0 | 0.12(0.00 to 0.24) | 29 | 0.06(-0.05 to 0.17) | 21 | 0.18(0.04 to 0.31) | 42 |
| Zero Life events (reference) |  |  |  |  |  |  |  |  |
| One Life event | 0.15(0.03 to 0.26) | 0 | 0.09(-0.03 to 0.21) | 13 | 0.05(-0.07 to 0.18) | 0 | 0.11(-0.03 to 0.31) | 41 |
| Two Life events | 0.25(0.12 to 0.38) | 0 | 0.14(0.01 to 0.27) | 0 | 0.06(-0.08 to 0.19) | 0 | 0.18(0.03 to 0.33) | 22 |
| Three or More Life events | 0.42(0.26 to 0.59) | 0 | 0.23(0.06 to 0.41) | 15 | 0.10(-0.08 to 0.28) | 0 | 0.31(0.14 to 0.47) | 0 |
| Arguments | 0.26(0.14 to 0.38) | 0 | 0.11(0.00 to 0.22) | 0 | 0.07(-0.04 to 0.18) | 0 | 0.17(0.06 to 0.29) | 0 |
| Bereavement | 0.08(-0.04 to 0.20) | 0 | 0.04(-0.07 to 0.15) | 0 | 0.01(-0.10 to 0.12) | 0 | 0.02(-0.09 to 0.14) | 0 |
| Debt | 0.27(0.11 to 0.42) | 50 | 0.13(-0.05 to 0.30) | 64 | 0.06(-0.11 to 0.23) | 57 | 0.22(0.04 to 0.39) | 63 |
| Divorce | 0.25(0.07 to 0.43) | 0 | 0.17(0.00 to 0.34) | 0 | 0.12(-0.05 to 0.30) | 0 | 0.24(0.06 to 0.41) | 0 |
| Victim of violent crime | 0.41(0.21 to 0.60) | 0 | 0.29(0.09 to 0.49) | 20 | 0.21(0.00 to 0.42) | 27 | 0.33(0.14 to 0.52) | 0 |
| Illness or Injury | -0.03(-0.23 to 0.17) | 69 | -0.04(-0.14 to 0.07) | 4 | -0.04(-0.14 to 0.06) | 0 | -0.04(-0.20 to 0.11) | 47 |
| Legal troubles | 0.16(-0.04 to 0.35) | 20 | 0.05(-0.17 to 0.28) | 48 | -0.02(-0.24 to 0.21) | 50 | 0.11(-0.11 to 0.33) | 40 |
| Sacked/Lost job | 0.05(-0.16 to 0.26) | 18 | 0.05(-0.13 to 0.24) | 3 | -0.08(-0.30 to 0.13) | 19 | 0.04(-0.15 to 0.23) | 0 |
| ^adjusted for allocated treatment, gender, and age; *adjusted for treatment, gender, age, ethnicity, baseline BDI-II score, average anxiety duration, depression duration, comorbid panic disorder, and history of antidepressant treatment; **‡**adjusted for treatment, gender, age, ethnicity, baseline BDI-II score, average anxiety duration, depression duration, comorbid panic disorder, history of antidepressant treatment, social support, marital status, employment status, and financial strain; ⸷adjusted for treatment, gender, age, ethnicity, average anxiety duration, depression duration, and history of antidepressant treatment | | | | | | | | |

**Supplementary Table 7.** Percentage differences in depressive symptoms at 3-4 months post-baseline per unit increase in life events variables, for participants with at least six months duration of depression at baseline (N=1910).

| **Life Events Variable** | **Adjusted for treatment, age, and gender^** | | **Additionally adjusted for depressive severity factors*** | | **Additionally adjusted for demographics and social support‡** | | **Removing factors temporally after the reported life events⸷** | |
| --- | --- | --- | --- | --- | --- | --- | --- | --- |
|  | **%(95%CI)** | **I^2^** | **%(95%CI)** | **I^2^** | **%(95%CI)** | **I^2^** | **%(95%CI)** | **I^2^** |
| Life events total score | 9.11(6.15 to 12.16) | 0 | 4.83(1.61 to 8.15) | 19 | 3.22(0.31 to 6.22) | 0 | 7.54(4.37 to 10.80) | 27 |
| Any life events | 16.27(7.83 to 25.38) | 0 | 7.96(-1.22 to 17.99) | 25 | 5.30(-2.64 to 13.89) | 4 | 15.24(7.96 to 23.02) | 0 |
| Zero Life events (reference) |  |  |  |  |  |  |  |  |
| One Life event | 9.13(0.07 to 19.02) | 0 | 4.93(-5.01 to 15.92) | 22 | 3.58(-6.90 to 15.24) | 25 | 8.97(1.03 to 17.54) | 0 |
| Two Life events | 18.41(7.60 to 30.32) | 0 | 10.63(0.24 to 22.09) | 5 | 7.22(-2.93 to 18.43) | 0 | 16.49(7.00 to 26.83) | 0 |
| Three or More Life events | 32.58(18.65 to 48.16) | 0 | 17.71(5.36 to 31.52) | 0 | 9.02(-4.18 to 24.05) | 0 | 30.25(18.37 to 43.33) | 0 |
| Arguments | 18.63(9.49 to 28.54) | 0 | 8.73(0.65 to 17.46) | 0 | 6.52(-1.32 to 14.98) | 0 | 16.50(8.54 to 25.04) | 0 |
| Bereavement | 6.62(-1.70 to 15.64) | 0 | 4.52(-3.13 to 12.77) | 0 | 2.30(-5.14 to 10.33) | 0 | 1.91(-5.21 to 9.58) | 0 |
| Debt | 18.34(4.76 to 33.67) | 56 | 8.26(-4.95 to 23.30) | 63 | 5.43(-7.16 to 19.73) | 52 | 21.24(9.03 to 34.82) | 59 |
| Divorce | 14.78(1.22 to 30.16) | 0 | 10.65(-1.88 to 24.78) | 0 | 7.37(-5.17 to 21.56) | 0 | 13.17(2.17 to 25.35) | 0 |
| Victim of violent crime | 33.76(18.49 to 51.00) | 0 | 22.75(9.97 to 37.03) | 0 | 16.61(1.00 to 34.63) | 30 | 24.21(10.77 to 39.28) | 0 |
| Illness or Injury | -5.41(-16.46 to 7.10) | 54 | -4.10(-10.83 to 3.13) | 0 | -4.54(-11.13 to 2.54) | 0 | -2.10(-11.19 to 7.93) | 48 |
| Legal troubles | 13.63(0.87 to 28.01) | 0 | 7.03(-4.08 to 19.44) | 0 | 3.02(-8.77 to 16.33) | 8 | 12.32(0.91 to 25.02) | 0 |
| Sacked/Lost job | 11.43(-5.10 to 30.83) | 15 | 10.21(-4.17 to 26.75) | 0 | -0.63(-14.63 to 15.66) | 8 | 1.04(-11.04 to 14.77) | 0 |
| ^adjusted for allocated treatment, gender, and age; *adjusted for treatment, gender, age, ethnicity, baseline BDI-II score, average anxiety duration, depression duration, comorbid panic disorder, and history of antidepressant treatment; **‡**adjusted for treatment, gender, age, ethnicity, baseline BDI-II score, average anxiety duration, depression duration, comorbid panic disorder, history of antidepressant treatment, social support, marital status, employment status, and financial strain; ⸷adjusted for treatment, gender, age, ethnicity, average anxiety duration, depression duration, and history of antidepressant treatment | | | | | | | | |

**Supplementary Table 8.** Differences in mean depressive symptoms at 3-4 months post-baseline per unit increase in life events variables, for participants with a first life-time depressive episode (N=610).

| **Life Events Variable** | **Adjusted for treatment, age, and gender^** | | **Additionally adjusted for depressive severity factors*** | | **Additionally adjusted for demographics and social support‡** | | **Removing factors temporally after the reported life events**^ | |
| --- | --- | --- | --- | --- | --- | --- | --- | --- |
|  | **Mean difference (95%CI)** | **I^2^** | **Mean difference (95%CI)** | **I^2^** | **Mean difference (95%CI)** | **I^2^** | **Mean difference (95%CI)** | **I^2^** |
| Life events total score | 0.10(0.04 to 0.17) | 0 | 0.06(-0.01 to 0.12) | 0 | 0.04(-0.03 to 0.11) | 0 | 0.10(0.04 to 0.17) | 0 |
| Any life events | 0.22(0.07 to 0.38) | 0 | 0.10(-0.05 to 0.25) | 0 | 0.08(-0.08 to 0.23) | 0 | 0.22(0.07 to 0.38) | 0 |
| Zero Life events (reference) |  |  |  |  |  |  |  |  |
| One Life event | 0.10(-0.09 to 0.28) | 0 | 0.04(-0.14 to 0.22) | 0 | 0.02(-0.18 to 0.21) | 0 | 0.10(-0.09 to 0.28) | 0 |
| Two Life events | 0.28(0.07 to 0.50) | 0 | 0.11(-0.10 to 0.32) | 0 | 0.05(-0.20 to 0.30) | 0 | 0.28(0.07 to 0.50) | 0 |
| Three or More Life events | 0.32(0.07 to 0.58) | 0 | 0.16(-0.10 to 0.42) | 0 | 0.21(-0.28 to 0.71) | 50 | 0.32(0.07 to 0.58) | 0 |
| Arguments | 0.23(0.04 to 0.42) | 0 | 0.19(0.01 to 0.37) | 0 | 0.15(-0.05 to 0.34) | 0 | 0.23(0.04 to 0.42) | 0 |
| Bereavement | 0.06(-0.13 to 0.24) | 0 | -0.04(-0.22 to 0.14) | 0 | -0.03(-0.22 to 0.15) | 4 | 0.06(-0.13 to 0.24) | 0 |
| Debt | 0.21(0.03 to 0.39) | 0 | 0.09(-0.08 to 0.26) | 3 | 0.08(-0.12 to 0.29) | 0 | 0.21(0.03 to 0.39) | 0 |
| Divorce | 0.12(-0.13 to 0.38) | 10 | 0.11(-0.1 to 0.33) | 0 | 0.10(-0.28 to 0.49) | 47 | 0.12(-0.13 to 0.38) | 10 |
| Victim of violent crime | 0.20(-0.15 to 0.55) | 0 | 0.23(-0.11 to 0.56) | 0 | 0.21(-0.13 to 0.55) | 0 | 0.20(-0.15 to 0.55) | 0 |
| Illness or Injury | -0.01(-0.2 to 0.19) | 16 | -0.04(-0.21 to 0.13) | 0 | -0.06(-0.23 to 0.12) | 0 | -0.01(-0.2 to 0.19) | 16 |
| Legal troubles | 0.31(-0.14 to 0.77) | 65 | 0.18(-0.12 to 0.47) | 28 | 0.19(-0.23 to 0.61) | 63 | 0.31(-0.14 to 0.77) | 65 |
| Sacked/Lost job | 0.35(-0.45 to 1.16) | 91 | 0.41-0.37 to 1.18) | 89 | 0.27(-0.47 to 1.01) | 79 | 0.35(-0.45 to 1.16) | 91 |
| ^adjusted for allocated treatment, gender, and age; *adjusted for treatment, gender, age, ethnicity, baseline BDI-II score, average anxiety duration, depression duration, and comorbid panic disorder; **‡**adjusted for treatment, gender, age, ethnicity, baseline BDI-II score, average anxiety duration, depression duration, comorbid panic disorder, social support, marital status, employment status, and financial strain. | | | | | | | | |

**Supplementary Table 9.** Percentage differences in depressive symptoms at 3-4 months post-baseline per unit increase in life events variables for participants with a first life-time depressive episode (N=610).

| **Life Events Variable** | **Adjusted for treatment, age, and gender^** | | **Additionally adjusted for depressive severity factors*** | | **Additionally adjusted for demographics and social support‡** | | **Removing factors temporally after the reported life events^** | |
| --- | --- | --- | --- | --- | --- | --- | --- | --- |
|  | **%(95%CI)** | **I^2^** | **%(95%CI)** | **I^2^** | **%(95%CI)** | **I^2^** | **%(95%CI)** | **I^2^** |
| Life events total score | 8.64(3.09 to 14.49) | 0 | 4.82(-0.53 to 10.45) | 0 | 4.04(-1.72 to 10.13) | 0 | 8.64(3.09 to 14.49) | 0 |
| Any life events | 13.56(-0.30 to 29.34) | 0 | 4.1(-8.23 to 18.09) | 0 | 2.06(-10.61 to 16.51) | 0 | 13.56(-0.30 to 29.34) | 0 |
| Zero Life events (reference) |  |  |  |  |  |  |  |  |
| One Life event | 4.02(-11.06 to 21.66) | 0 | -2.29(-16.01 to 13.67) | 0 | -2.97(-17.67 to 14.35) | 0 | 4.02(-11.06 to 21.66) | 0 |
| Two Life events | 21.14(1.83 to 44.11) | 0 | 7.35(-9.35 to 27.12) | 0 | 0.25(-18.09 to 22.69) | 0 | 21.14(1.83 to 44.11) | 0 |
| Three or More Life events | 34.39(10.75 to 63.08) | 0 | 19.17(-4.80 to 49.18) | 0 | 13.39(-14.76 to 50.85) | 4 | 34.39(10.75 to 63.08) | 0 |
| Arguments | 22.39(4.90 to 42.79) | 0 | 18.14(1.33 to 37.73) | 13 | 14.17(-2.28 to 33.39) | 0 | 22.39(4.90 to 42.79) | 0 |
| Bereavement | 15.36(0.47 to 32.46) | 0 | 3.98(-9.84 to 19.93) | 0 | 4.60(-10.24 to 21.90) | 0 | 15.36(0.47 to 32.46) | 0 |
| Debt | 13.26(-5.08 to 35.14) | 29 | 4.72(-10.93 to 23.13) | 21 | 1.78(-13.88 to 20.29) | 0 | 13.26(-5.08 to 35.14) | 29 |
| Divorce | 15.38(-6.53 to 42.43) | 0 | 13.39(-6.49 to 37.51) | 0 | 11.82(-13.74 to 44.95) | 21 | 15.38(-6.53 to 42.43) | 0 |
| Victim of violent crime | 17.88(-9.67 to 53.84) | 0 | 17.50(-9.26 to 52.14) | 0 | 15.88(-11.32 to 51.42) | 0 | 17.88(-9.67 to 53.84) | 0 |
| Illness or Injury | 1.84(-12.12 to 18.02) | 0 | -2.50(-15.98 to 13.14) | 0 | -3.87(-17.40 to 11.89) | 0 | 1.84(-12.12 to 18.02) | 0 |
| Legal troubles | 26.79(-2.72 to 65.27) | 48 | 25.36(5.58 to 48.85) | 3 | 20.83(-6.43 to 56.03) | 43 | 26.79(-2.72 to 65.27) | 48 |
| Sacked/Lost job | 18.65(-25.2 to 88.21) | 81 | 24.99(-19.15 to 93.24) | 76 | 16.59(-28.58 to 90.35) | 70 | 18.65(-25.2 to 88.21) | 81 |
| ^adjusted for allocated treatment, gender, and age; *adjusted for treatment, gender, age, ethnicity, baseline BDI-II score, average anxiety duration, depression duration, and comorbid panic disorder; **‡**adjusted for treatment, gender, age, ethnicity, baseline BDI-II score, average anxiety duration, depression duration, comorbid panic disorder, social support, marital status, employment status, and financial strain. | | | | | | | | |

**Supplementary Table 10.** Odds ratios for remission at 3-4 months post-baseline per unit increase in life events variables, across the whole sample (N=2858).

| **Life Events Variable** | **Adjusted for treatment, age, and gender^** | | **Additionally adjusted for depressive severity factors*** | | **Additionally adjusted for demographics and social support‡** | | **Removing factors temporally after the reported life events⸷** | |
| --- | --- | --- | --- | --- | --- | --- | --- | --- |
|  | **OR(95%CI)** | **I^2^** | **OR(95%CI)** | **I^2^** | **OR(95%CI)** | **I^2^** | **OR(95%CI)** | **I^2^** |
| Life events total score | 0.84(0.76 to 0.93) | 45 | 0.91(0.82 to 1.01) | 34 | 0.92(0.83 to 1.02) | 31 | 0.84(0.76 to 0.94) | 44 |
| Any life events | 0.70(0.58 to 0.84) | 0 | 0.81(0.65 to 1.02) | 18 | 0.82(0.66 to 1.03) | 17 | 0.72(0.59 to 0.86) | 0 |
| Zero Life events (reference) |  |  |  |  |  |  |  |  |
| One Life event | 0.84(0.68 to 1.03) | 0 | 0.88(0.70 to 1.11) | 0 | 0.88(0.70 to 1.11) | 0 | 0.84(0.68 to 1.05) | 0 |
| Two Life events | 0.66(0.51 to 0.87) | 18 | 0.77(0.57 to 1.03) | 21 | 0.79(0.58 to 1.07) | 27 | 0.68(0.52 to 0.89) | 17 |
| Three or More Life events | 0.53(0.39 to 0.71) | 0 | 0.68(0.49 to 0.93) | 0 | 0.69(0.50 to 0.95) | 0 | 0.53(0.40 to 0.72) | 0 |
| Arguments | 0.64(0.52 to 0.79) | 0 | 0.78(0.62 to 0.98) | 0 | 0.83(0.66 to 1.05) | 0 | 0.65(0.53 to 0.80) | 0 |
| Bereavement | 0.93(0.74 to 1.19) | 16 | 0.97(0.75 to 1.27) | 22 | 0.99(0.75 to 1.29) | 20 | 0.95(0.74 to 1.21) | 17 |
| Debt | 0.70(0.54 to 0.91) | 46 | 0.84(0.64 to 1.09) | 41 | 0.88(0.68 to 1.13) | 33 | 0.71(0.55 to 0.92) | 43 |
| Divorce | 0.72(0.54 to 0.95) | 0 | 0.76(0.56 to 1.03) | 0 | 0.81(0.59 to 1.10) | 0 | 0.71(0.54 to 0.94) | 0 |
| Victim of violent crime | 0.62(0.44 to 0.88) | 0 | 0.74(0.52 to 1.06) | 0 | 0.82(0.57 to 1.19) | 0 | 0.63(0.44 to 0.90) | 0 |
| Illness or Injury | 1.16(0.91 to 1.49) | 41 | 1.16(0.95 to 1.41) | 0 | 1.15(0.94 to 1.42) | 0 | 1.16(0.90 to 1.48) | 40 |
| Legal troubles | 0.83(0.61 to 1.14) | 0 | 0.97(0.68 to 1.38) | 12 | 1.08(0.75 to 1.56) | 8 | 0.85(0.62 to 1.17) | 0 |
| Sacked/Lost job | 0.94(0.65 to 1.36) | 0 | 0.96(0.65 to 1.41) | 0 | 1.01(0.68 to 1.50) | 0 | 0.98(0.67 to 1.43) | 0 |
| ^adjusted for allocated treatment, gender, and age; *adjusted for treatment, gender, age, ethnicity, baseline BDI-II score, average anxiety duration, depression duration, comorbid panic disorder, and history of antidepressant treatment; **‡**adjusted for treatment, gender, age, ethnicity, baseline BDI-II score, average anxiety duration, depression duration, comorbid panic disorder, history of antidepressant treatment, social support, marital status, employment status, and financial strain; ⸷adjusted for treatment, gender, age, ethnicity, average anxiety duration, depression duration, and history of antidepressant treatment | | | | | | | | |

**Supplementary Table 11.** Odds ratios for remission at 3-4 months post-baseline per unit increase in life events variables, for participants with at least six months duration of depression at baseline (N=1910).

| **Life Events Variable** | **Adjusted for treatment, age, and gender^** | | **Additionally adjusted for depressive severity factors*** | | **Additionally adjusted for demographics and social support‡** | | **Removing factors temporally after the reported life events⸷** | |
| --- | --- | --- | --- | --- | --- | --- | --- | --- |
|  | **OR(95%CI)** | **I^2^** | **OR(95%CI)** | **I^2^** | **OR(95%CI)** | **I^2^** | **OR(95%CI)** | **I^2^** |
| Life events total score | 0.794(0.70 to 0.89) | 31 | 0.86(0.76 to 0.97) | 31 | 0.89(0.78 to 1.02) | 33 | 0.83(0.74 to 0.93) | 24 |
| Any life events | 0.68(0.54 to 0.85) | 0 | 0.78(0.61 to 0.99) | 0 | 0.83(0.63 to 1.11) | 12 | 0.75(0.59 to 0.94) | 0 |
| Zero Life events (reference) |  |  |  |  |  |  |  |  |
| One Life event | 0.86(0.67 to 1.11) | 0 | 0.90(0.68 to 1.19) | 0 | 0.95(0.69 to 1.32) | 13 | 0.90(0.69 to 1.17) | 0 |
| Two Life events | 0.59(0.43 to 0.79) | 0 | 0.63(0.45 to 0.88) | 0 | 0.67(0.45 to 0.99) | 8 | 0.62(0.44 to 0.86) | 0 |
| Three or More Life events | 0.51(0.33 to 0.78) | 21 | 0.64(0.42 to 0.98) | 6 | 0.70(0.43 to 1.15) | 0 | 0.60(0.40 to 0.89) | 0 |
| Arguments | 0.56(0.43 to 0.73) | 0 | 0.67(0.50 to 0.89) | 0 | 0.61(0.51 to 0.93) | 0 | 0.63(0.47 to 0.83) | 0 |
| Bereavement | 0.79(0.60 to 1.03) | 0 | 0.83(0.63 to 1.10) | 0 | 0.86(0.65 to 1.15) | 0 | 0.85(0.64 to 1.12) | 0 |
| Debt | 0.75(0.55 to 1.02) | 41 | 0.90(0.65 to 1.24) | 36 | 0.99(0.69 to 1.42) | 32 | 0.82(0.60 to 1.13) | 41 |
| Divorce | 0.65(0.44 to 0.95) | 0 | 0.70(0.46 to 1.07) | 0 | 0.73(0.47 to 1.14) | 0 | 0.64(0.43 to 0.95) | 0 |
| Victim of violent crime | 0.48(0.29 to 0.77) | 0 | 0.55(0.32 to 0.92) | 7 | 0.56(0.31 to 1.02) | 21 | 0.53(0.32 to 0.85) | 0 |
| Illness or Injury | 1.21(0.86 to 1.69) | 49 | 1.24(0.97 to 1.58) | 0 | 1.25(0.97 to 1.60) | 0 | 1.26(0.97 to 1.63) | 14 |
| Legal troubles | 0.79(0.50 to 1.25) | 21 | 0.86(0.51 to 1.44) | 33 | 0.95(0.56 to 1.62) | 30 | 0.83(0.51 to 1.36) | 27 |
| Sacked/Lost job | 0.80(0.48 to 1.31) | 0 | 0.83(0.49 to 1.40) | 0 | 1.11(0.64 to 1.94) | 0 | 0.81(0.48 to 1.37) | 0 |
| ^adjusted for allocated treatment, gender, and age; *adjusted for treatment, gender, age, ethnicity, baseline BDI-II score, average anxiety duration, depression duration, comorbid panic disorder, and history of antidepressant treatment; **‡**adjusted for treatment, gender, age, ethnicity, baseline BDI-II score, average anxiety duration, depression duration, comorbid panic disorder, history of antidepressant treatment, social support, marital status, employment status, and financial strain; ⸷adjusted for treatment, gender, age, ethnicity, average anxiety duration, depression duration, and history of antidepressant treatment | | | | | | | | |

**Supplementary Table 12.** Odds ratios for remission at 3-4 months post-baseline per unit increase in life events variables, for participants with a first life-time depressive episode (N=610).

| **Life Events Variable** | **Adjusted for treatment, age, and gender^** | | **Additionally adjusted for depressive severity factors*** | | **Additionally adjusted for demographics and social support‡** | | **Removing factors temporally after the reported life events^** | |
| --- | --- | --- | --- | --- | --- | --- | --- | --- |
|  | **OR(95%CI)** | **I^2^** | **OR(95%CI)** | **I^2^** | **OR(95%CI)** | **I^2^** | **OR(95%CI)** | **I^2^** |
| Life events total score | 0.86(0.73 to 1.01) | 0 | 0.93(0.78 to 1.11) | 0 | 0.97(0.8 to 1.19) | 0 | 0.86(0.73 to 1.01) | 0 |
| Any life events | 0.92(0.61 to 1.37) | 0 | 1.05(0.66 to 1.65) | 0 | 1.20(0.72 to 2.00) | 0 | 0.92(0.61 to 1.37) | 0 |
| Zero Life events (reference) |  |  |  |  |  |  |  |  |
| One Life event | 1.16(0.71 to 1.89) | 0 | 1.28(0.73 to 2.24) | 0 | 1.25(0.71 to 2.19) | 0 | 1.16(0.71 to 1.89) | 0 |
| Two Life events | 0.77(0.42 to 1.42) | 14 | 1.04(0.55 to 1.95) | 0 | 1.35(0.60 to 3.01) | 0 | 0.77(0.42 to 1.42) | 14 |
| Three or More Life events | 0.66(0.33 to 1.34) | 0 | 0.85(0.41 to 1.78) | 0 | 1.04(0.45 to 2.44) | 0 | 0.66(0.33 to 1.34) | 0 |
| Arguments | 0.62(0.38 to 1.01) | 0 | 0.75(0.44 to 1.29) | 0 | 0.68(0.38 to 1.23) | 0 | 0.62(0.38 to 1.01) | 0 |
| Bereavement | 1.01(0.59 to 1.75) | 0 | 1.11(0.60 to 2.04) | 0 | 1.00(0.53 to 1.89) | 0 | 1.01(0.59 to 1.75) | 0 |
| Debt | 0.85(0.55 to 1.31) | 0 | 0.92(0.57 to 1.49) | 0 | 1.07(0.59 to 1.92) | 0 | 0.85(0.55 to 1.31) | 0 |
| Divorce | 0.77(0.43 to 1.35) | 0 | 0.81(0.43 to 1.51) | 0 | 0.98(0.47 to 2.06) | 0 | 0.77(0.43 to 1.35) | 0 |
| Victim of violent crime | 0.74(0.33 to 1.68) | 0 | 0.85(0.35 to 2.07) | 0 | 0.77(0.30 to 1.95) | 0 | 0.74(0.33 to 1.68) | 0 |
| Illness or Injury | 1.40(0.91 to 2.17) | 0 | 1.51(0.93 to 2.47) | 0 | 1.69(0.99 to 2.87) | 0 | 1.40(0.91 to 2.17) | 0 |
| Legal troubles | 0.95(0.47 to 1.90) | 0 | 0.95(0.45 to 2.02) | 0 | 1.00(0.44 to 2.29) | 0 | 0.95(0.47 to 1.90) | 0 |
| Sacked/Lost job | 1.33(0.62 to 2.85) | 0 | 1.13(0.49 to 2.62) | 0 | 1.27(0.43 to 3.70) | 10 | 1.33(0.62 to 2.85) | 0 |
| ^adjusted for allocated treatment, gender, and age; *adjusted for treatment, gender, age, ethnicity, baseline BDI-II score, average anxiety duration, depression duration, and comorbid panic disorder; **‡**adjusted for treatment, gender, age, ethnicity, baseline BDI-II score, average anxiety duration, depression duration, comorbid panic disorder, social support, marital status, employment status, and financial strain. | | | | | | | | |

**Supplementary Table 13.** Differences in mean depressive symptoms at 6-8 months post-baseline per unit increase in life events variables, across the whole sample (N=1598).

| **Life Events Variable** | **Adjusted for treatment, age, and gender^** | | **Additionally adjusted for depressive severity factors*** | | **Additionally adjusted for demographics and social support‡** | | **Removing factors temporally after the reported life events⸷** | |
| --- | --- | --- | --- | --- | --- | --- | --- | --- |
|  | **Mean difference (95%CI)** | **I^2^** | **Mean difference (95%CI)** | **I^2^** | **Mean difference (95%CI)** | **I^2^** | **Mean difference (95%CI)** | **I^2^** |
| Life events total score | 0.11(0.06 to 0.16) | 0 | 0.04(-0.01 to 0.09) | 0 | 0.00(-0.05 to 0.05) | 0 | 0.10(0.05 to 0.15) | 0 |
| Any life events | 0.29(0.17 to 0.40) | 0 | 0.17(0.06 to 0.28) | 0 | 0.11(0.00 to 0.22) | 0 | 0.28(0.17 to 0.40) | 0 |
| Zero Life events (reference) |  |  |  |  |  |  |  |  |
| One Life event | 0.27(0.13 to 0.40) | 0 | 0.21(0.09 to 0.34) | 0 | 0.19(0.06 to 0.31) | 0 | 0.27(0.14 to 0.40) | 0 |
| Two Life events | 0.21(0.06 to 0.37) | 0 | 0.08(-0.06 to 0.23) | 0 | 0.00(-0.15 to 0.15) | 0 | 0.21(0.05 to 0.36) | 0 |
| Three or More Life events | 0.42(0.23 to 0.61) | 0 | 0.26(0.08 to 0.45) | 0 | 0.12(-0.08 to 0.33) | 0 | 0.42(0.23 to 0.61) | 0 |
| Arguments | 0.29(0.14 to 0.44) | 14 | 0.12(-0.01 to 0.25) | 0 | 0.08(-0.04 to 0.21) | 0 | 0.27(0.11 to 0.43) | 26 |
| Bereavement | -0.05(-0.20 to 0.09) | 0 | -0.05(-0.19 to 0.08) | 0 | -0.08(-0.21 to 0.06) | 0 | -0.06(-0.20 to 0.08) | 0 |
| Debt | 0.26(0.09 to 0.43) | 45 | 0.14(0.02 to 0.26) | 7 | 0.03(-0.10 to 0.16) | 0 | 0.25(0.09 to 0.42) | 43 |
| Divorce | -0.02(-0.21 to 0.18) | 0 | -0.09(-0.28 to 0.09) | 0 | -0.19(-0.31 to 0.01) | 0 | -0.01(-0.21 to 0.18) | 0 |
| Victim of violent crime | 0.24(0.00 to 0.49) | 0 | 0.14(-0.08 to 0.36) | 0 | 0.09(-0.13 to 0.31) | 0 | 0.22(-0.02 to 0.46) | 0 |
| Illness or Injury | 0.04(-0.11 to 0.20) | 30 | 0.01(-0.12 to 0.15) | 16 | 0.01(-0.13 to 0.14) | 16 | 0.05(-0.10 to 0.19) | 20 |
| Legal troubles | 0.16(-0.11 to 0.43) | 33 | -0.01(-0.32 to 0.31) | 55 | -0.06(-0.39 to 0.26) | 59 | 0.15(-0.13 to 0.43) | 36 |
| Sacked/Lost job | 0.29(-0.06 to 0.63) | 53 | 0.30(0.03 to 0.58) | 43 | 0.18(-0.11 to 0.48) | 39 | 0.28(-0.05 to 0.61) | 49 |
| ^adjusted for allocated treatment, gender, and age; *adjusted for treatment, gender, age, ethnicity, baseline BDI-II score, average anxiety duration, depression duration, comorbid panic disorder, and history of antidepressant treatment; **‡**adjusted for treatment, gender, age, ethnicity, baseline BDI-II score, average anxiety duration, depression duration, comorbid panic disorder, history of antidepressant treatment, social support, marital status, employment status, and financial strain; ⸷adjusted for treatment, gender, age, ethnicity, average anxiety duration, depression duration, and history of antidepressant treatment | | | | | | | | |

**Supplementary Table 14.** Differences in mean depressive symptoms at 6-8 months post-baseline per unit increase in life events variables, for participants with at least six months duration of depression at baseline (N=1183).

| **Life Events Variable** | **Adjusted for treatment, age, and gender^** | | **Additionally adjusted for depressive severity factors*** | | **Additionally adjusted for demographics and social support‡** | | **Removing factors temporally after the reported life events⸷** | |
| --- | --- | --- | --- | --- | --- | --- | --- | --- |
|  | **Mean difference (95%CI)** | **I^2^** | **Mean difference (95%CI)** | **I^2^** | **Mean difference (95%CI)** | **I^2^** | **Mean difference (95%CI)** | **I^2^** |
| Life events total score | 0.11(0.05 to 0.17) | 0 | 0.04(-0.01 to 0.10) | 0 | 0.00(-0.06 to 0.05) | 0 | 0.09(0.03 to 0.15) | 0 |
| Any life events | 0.29(0.15 to 0.43) | 0 | 0.16(0.03 to 0.29) | 0 | 0.10(-0.03 to 0.23) | 0 | 0.26(0.13 to 0.39) | 0 |
| Zero Life events (reference) |  |  |  |  |  |  |  |  |
| One Life event | 0.26(0.11 to 0.42) | 0 | 0.20(0.05 to 0.34) | 0 | 0.16(0.01 to 0.31) | 0 | 0.25(0.10 to 0.41) | 0 |
| Two Life events | 0.25(0.07 to 0.44) | 0 | 0.10(-0.07 to 0.28) | 0 | 0.00(-0.19 to 0.18) | 0 | 0.20(0.02 to 0.38) | 0 |
| Three or More Life events | 0.39(0.17 to 0.62) | 0 | 0.23(0.01 to 0.45) | 0 | 0.04(-0.19 to 0.18) | 0 | 0.35(0.13 to 0.57) | 0 |
| Arguments | 0.35(0.19 to 0.52) | 0 | 0.18(0.02 to 0.33) | 0 | 0.14(-0.01 to 0.29) | 0 | 0.29(0.13 to 0.45) | 0 |
| Bereavement | 0.00(-0.17 to 0.17) | 0 | -0.02(-0.18 to 0.13) | 0 | -0.04(-0.25 to 0.16) | 0 | -0.03(-0.20 to 0.14) | 7 |
| Debt | 0.24(0.06 to 0.42) | 30 | 0.11(-0.03 to 0.24) | 0 | -0.01(-0.16 to 0.14) | 33 | 0.23(0.05 to 0.41) | 35 |
| Divorce | -0.03(-0.27 to 0.21) | 0 | -0.11(-0.34 to 0.11) | 0 | -0.20(-0.44 to 0.04) | 0 | -0.04(-0.28 to 0.20) | 0 |
| Victim of violent crime | 0.34(0.06 to 0.61) | 0 | 0.20(-0.06 to 0.45) | 0 | 0.11(-0.14 to 0.36) | 0 | 0.28(0.01 to 0.54) | 0 |
| Illness or Injury | 0.02(-0.13 to 0.18) | 0 | -0.06(-0.24 to 0.13) | 35 | -0.07(-0.23 to 0.10) | 20 | -0.04(-0.22 to 0.14) | 24 |
| Legal troubles | 0.07(-0.19 to 0.34) | 13 | -0.06(-0.35 to 0.24) | 37 | -0.13(-0.44 to 0.19) | 42 | 0.05(-0.24 to 0.33) | 25 |
| Sacked/Lost job | 0.37(-0.06 to 0.80) | 58 | 0.40(0.09 to 0.71) | 33 | 0.27(-0.04 to 0.58) | 22 | 0.40(0.03 to 0.77) | 47 |
| ^adjusted for allocated treatment, gender, and age; *adjusted for treatment, gender, age, ethnicity, baseline BDI-II score, average anxiety duration, depression duration, comorbid panic disorder, and history of antidepressant treatment; **‡**adjusted for treatment, gender, age, ethnicity, baseline BDI-II score, average anxiety duration, depression duration, comorbid panic disorder, history of antidepressant treatment, social support, marital status, employment status, and financial strain; ⸷adjusted for treatment, gender, age, ethnicity, average anxiety duration, depression duration, and history of antidepressant treatment | | | | | | | | |

**Supplementary Table 15.** Differences in mean depressive symptoms at 6-8 months post-baseline per unit increase in life events variables, for participants with a first life-time depressive episode (N=313).

| **Life Events Variable** | **Adjusted for treatment, age, and gender^** | | **Additionally adjusted for depressive severity factors*** | | **Additionally adjusted for demographics and social support‡** | | **Removing factors temporally after the reported life events^** | |
| --- | --- | --- | --- | --- | --- | --- | --- | --- |
|  | **Mean difference (95%CI)** | **I^2^** | **Mean difference (95%CI)** | **I^2^** | **Mean difference (95%CI)** | **I^2^** | 0.05(-0.05 to 0.14) | 0 |
| Life events total score | 0.05(-0.05 to 0.14) | 0 | -0.02(-0.12 to 0.09) | 0 | -0.06(-0.19 to 0.06) | 0 | 0.16(-0.09 to 0.41) | 0 |
| Any life events | 0.16(-0.09 to 0.41) | 0 | 0.01(-0.22 to 0.24) | 0 | -0.04(-0.30 to 0.22) | 0 |  |  |
| Zero Life events (reference) |  |  |  |  |  |  | 0.16(-0.14 to 0.46) | 0 |
| One Life event | 0.16(-0.14 to 0.46) | 0 | 0.02(-0.25 to 0.30) | 0 | -0.03(-0.34 to 0.28) | 0 | 0.09(-0.27 to 0.44) | 0 |
| Two Life events | 0.09(-0.27 to 0.44) | 0 | -0.09(-0.43 to 0.24) | 0 | -0.12(-0.54 to 0.30) | 0 | 0.21(-0.19 to 0.62) | 0 |
| Three or More Life events | 0.21(-0.19 to 0.62) | 0 | 0.08(-0.38 to 0.54) | 0 | 0.01(-0.67 to 0.69) | 0 | 0.00(-0.26 to 0.27) | 0 |
| Arguments | 0.00(-0.26 to 0.27) | 0 | -0.13(-0.52 to 0.26) | 44 | -0.17(-0.58 to 0.24) | 41 | 0.11(-0.20 to 0.41) | 0 |
| Bereavement | 0.11(-0.20 to 0.41) | 0 | -0.02(-0.33 to 0.28) | 0 | -0.08(-0.41 to 0.25) | 0 | 0.15(-0.14 to 0.43) | 11 |
| Debt | 0.15(-0.14 to 0.43) | 11 | 0.05(-0.21 to 0.31) | 0 | -0.06(-0.45 to 0.34) | 25 | -0.31(-0.76 to 0.13) | 58 |
| Divorce | -0.31(-0.76 to 0.13) | 58 | -0.29(-0.64 to 0.07) | 0 | -0.40(-0.85 to 0.06) | 0 | -0.29(-0.78 to 0.20) | 0 |
| Victim of violent crime | -0.29(-0.78 to 0.20) | 0 | -0.24(-0.77 to 0.29) | 0 | -0.24(-0.81 to 0.32) | 0 | -0.04(-0.38 to 0.30) | 22 |
| Illness or Injury | -0.04(-0.38 to 0.30) | 22 | -0.15(-0.42 to 0.13) | 0 | -0.15(-0.45 to 0.14) | 0 | 0.19(-0.29 to 0.67) | 37 |
| Legal troubles | 0.19(-0.29 to 0.67) | 37 | 0.00(-0.40 to 0.40) | 0 | -0.01(-0.44 to 0.42) | 0 | 0.40(-0.23 to 1.03) | 75 |
| Sacked/Lost job | 0.40(-0.23 to 1.03) | 75 | 0.47(-0.16 to 1.11) | 72 | 0.41(-0.31 to 1.12) | 55 | 0.05(-0.05 to 0.14) | 0 |
| ^adjusted for allocated treatment, gender, and age; *adjusted for treatment, gender, age, ethnicity, baseline BDI-II score, average anxiety duration, depression duration, and comorbid panic disorder; **‡**adjusted for treatment, gender, age, ethnicity, baseline BDI-II score, average anxiety duration, depression duration, comorbid panic disorder, social support, marital status, employment status, and financial strain. | | | | | | | | |

**Supplementary Table 16.** Differences in mean BDI-II scores at 3-4 months post-baseline per unit increase in life events variables, across the whole sample (N=2380).

| **Life Events Variable** | **Adjusted for treatment, age, and gender^** | | **Additionally adjusted for depressive severity factors*** | | **Additionally adjusted for demographics and social support‡** | | **Removing factors temporally after the reported life events⸷** | |
| --- | --- | --- | --- | --- | --- | --- | --- | --- |
|  | **Mean difference (95%CI)** | **I^2^** | **Mean difference (95%CI)** | **I^2^** | **Mean difference (95%CI)** | **I^2^** | **Mean difference (95%CI)** | **I^2^** |
| Life events total score | 1.23(0.79 to 1.66) | 0 | 0.42(-0.05 to 0.89) | 20 | 0.11(-0.32 to 0.55) | 0 | 1.14(0.70 to 1.57) | 0 |
| Any life events | 2.48(1.44 to 3.51) | 0 | 0.96(-0.12 to 2.04) | 17 | 0.40(-0.59 to 1.39) | 0 | 2.30(1.26 to 3.34) | 0 |
| Zero Life events (reference) |  |  |  |  |  |  |  |  |
| One Life event | 1.36(0.16 to 2.56) | 0 | 0.66(-0.49 to 1.81) | 8 | 0.45(-0.66 to 1.56) | 0 | 1.30(0.10 to 2.50) | 0 |
| Two Life events | 2.34(0.96 to 3.71) | 0 | 0.75(-0.57 to 2.08) | 0 | 0.14(-1.23 to 1.51) | 0 | 2.20(0.82 to 3.57) | 0 |
| Three or More Life events | 4.54(2.87 to 6.21) | 0 | 1.95(0.21 to 3.69) | 13 | 0.90(-0.82 to 2.61) | 0 | 4.28(2.61 to 5.95) | 0 |
| Arguments | 2.69(1.45 to 3.92) | 14 | 1.05(-0.09 to 2.19) | 0 | 0.79(-0.37 to 1.95) | 0 | 2.47(1.24 to 3.71) | 0 |
| Bereavement | 0.27(-1.06 to 1.59) | 0 | -0.19(-1.36 to 2.19) | 0 | -0.41(-1.59 to 0.77) | 0 | 0.17(-1.14 to 1.47) | 0 |
| Debt | 3.15(1.49 to 4.81) | 45 | 1.17(-0.51 to 2.85) | 58 | 0.49(-1.00 to 1.98) | 35 | 2.98(1.36 to 4.59) | 48 |
| Divorce | 2.06(0.39 to 3.73) | 0 | 1.23(-0.75 to 3.21) | 37 | 0.35(-1.90 to 2.61) | 45 | 2.07(0.40 to 3.74) | 0 |
| Victim of violent crime | 3.34(0.94 to 5.74) | 0 | 2.01(-0.30 to 4.33) | 29 | 1.53(-0.38 to 3.43) | 0 | 3.11(0.77 to 5.45) | 22 |
| Illness or Injury | -0.06(-1.88 to 1.75) | 30 | 0.05(-1.10 to 1.19) | 18 | 0.18(-1.07 to 1.44) | 30 | 0.00(-1.79 to 1.79) | 58 |
| Legal troubles | 0.72(-1.18 to 1.75) | 33 | -0.74(-2.49 to 1.00) | 3 | -1.35(-3.04 to 0.35) | 0 | 0.51(-1.38 to 2.40) | 0 |
| Sacked/Lost job | -1.26(-3.12 to 0.60) | 53 | -1.21(-2.98 to 0.57) | 0 | -2.58(-4.53 to -0.62) | 1 | -1.47(-3.35 to 0.40) | 0 |
| ^adjusted for allocated treatment, gender, and age; *adjusted for treatment, gender, age, ethnicity, baseline BDI-II score, average anxiety duration, depression duration, comorbid panic disorder, and history of antidepressant treatment; **‡**adjusted for treatment, gender, age, ethnicity, baseline BDI-II score, average anxiety duration, depression duration, comorbid panic disorder, history of antidepressant treatment, social support, marital status, employment status, and financial strain; ⸷adjusted for treatment, gender, age, ethnicity, average anxiety duration, depression duration, and history of antidepressant treatment | | | | | | | | |

**Supplementary Table 17.** Differences in mean BDI-II scores at 3-4 months post-baseline per unit increase in life events variables, for participants with at least six months duration of depression at baseline (N=1482).

| **Life Events Variable** | **Adjusted for treatment, age, and gender^** | | **Additionally adjusted for depressive severity factors*** | | **Additionally adjusted for demographics and social support‡** | | **Removing factors temporally after the reported life events⸷** | |
| --- | --- | --- | --- | --- | --- | --- | --- | --- |
|  | **Mean difference (95%CI)** | **I^2^** | **Mean difference (95%CI)** | **I^2^** | **Mean difference (95%CI)** | **I^2^** | **Mean difference (95%CI)** | **I^2^** |
| Life events total score | 1.48(0.93 to 2.03) | 0 | 0.61(-0.02 to 1.24) | 27 | 0.29(-0.36 to 0.94) | 26 | 0.98(0.43 to 1.52) | 0 |
| Any life events | 2.38(1.02 to 3.74) | 0 | 0.79(-0.49 to 2.07) | 0 | 0.26(-1.06 to 1.57) | 0 | 1.52(-0.08 to 3.11) | 24 |
| Zero Life events (reference) |  |  |  |  |  |  |  |  |
| One Life event | 1.23(-0.34 to 2.81) | 0 | 0.44(-0.99 to 1.87) | 0 | 0.08(-1.39 to 1.56) | 0 | 0.67(-1.19 to 2.52) | 25 |
| Two Life events | 1.23(0.49 to 4.12) | 0 | 0.81(-0.96 to 2.58) | 0 | 0.17(-1.67 to 2.00) | 0 | 1.44(-0.40 to 3.27) | 0 |
| Three or More Life events | 4.65(2.41 to 6.89) | 0 | 2.11(-0.06 to 4.28) | 0 | 0.81(-1.57 to 3.20) | 0 | 2.93(0.65 to 5.22) | 0 |
| Arguments | 3.23(1.60 to 4.87) | 0 | 1.55(0.07 to 3.04) | 0 | 1.17(-0.31 to 2.65) | 0 | 2.13(0.54 to 3.71) | 0 |
| Bereavement | 1.23(-0.48 to 2.93) | 0 | 0.55(-0.96 to 2.06) | 0 | 0.19(-1.32 to 1.71) | 0 | 0.42(-1.21 to 2.05) | 0 |
| Debt | 2.69(0.49 to 4.88) | 53 | 0.65(-1.51 to 2.81) | 55 | -0.12(-2.09 to 1.85) | 38 | 1.84(-0.58 to 4.27) | 62 |
| Divorce | 2.71(0.34 to 5.08) | 0 | 1.80(-0.33 to 3.94) | 0 | 1.28(-0.99 to 3.55) | 0 | 2.78(0.43 to 5.12) | 0 |
| Victim of violent crime | 4.57(2.01 to 7.13) | 0 | 3.24(0.30 to 6.19) | 30 | 2.57(-0.60 to 5.73) | 36 | 3.68(1.19 to 6.17) |  |
| Illness or Injury | -0.53(-3.33 to 2.28) | 70 | -0.55(-1.89 to 0.78) | 0 | -0.47(-1.80 to 0.87) | 0 | -0.52(-2.63 to 1.59) | 49 |
| Legal troubles | 0.42(-1.96 to 2.81) | 0 | -0.62(-3.35 to 2.12) | 36 | -1.48(-4.14 to 1.19) | 33 | -0.29(-2.57 to 2.00) | 1 |
| Sacked/Lost job | -0.40(-2.85 to 1.25) | 0 | -0.21(-2.52 to 2.11) | 0 | -1.69(-4.44 to -1.07) | 14 | -0.37(-2.81 to 2.08) | 0 |
| ^adjusted for allocated treatment, gender, and age; *adjusted for treatment, gender, age, ethnicity, baseline BDI-II score, average anxiety duration, depression duration, comorbid panic disorder, and history of antidepressant treatment; **‡**adjusted for treatment, gender, age, ethnicity, baseline BDI-II score, average anxiety duration, depression duration, comorbid panic disorder, history of antidepressant treatment, social support, marital status, employment status, and financial strain; ⸷adjusted for treatment, gender, age, ethnicity, average anxiety duration, depression duration, and history of antidepressant treatment | | | | | | | | |

**Supplementary Table 18.** Differences in mean BDI-II scores at 3-4 months post-baseline per unit increase in life events variables, for participants with a first life-time depressive episode (N=556).

| **Life Events Variable** | **Adjusted for treatment, age, and gender^** | | **Additionally adjusted for depressive severity factors*** | | **Additionally adjusted for demographics and social support‡** | | **Removing factors temporally after the reported life events^** | |
| --- | --- | --- | --- | --- | --- | --- | --- | --- |
|  | **Mean difference (95%CI)** | **I^2^** | **Mean difference (95%CI)** | **I^2^** | **Mean difference (95%CI)** | **I^2^** | **Mean difference (95%CI)** | **I^2^** |
| Life events total score | 1.10(0.30 to 1.90) | 0 | 0.63(-0.13 to 1.40) | 0 | 0.44(-0.41 to 1.28) | 0 | 1.10(0.30 to 1.90) | 0 |
| Any life events | 2.55(0.70 to 4.41) | 0 | 1.24(-0.56 to 3.04) | 1 | 0.90(-0.99 to 2.78) | 0 | 2.55(0.70 to 4.41) | 0 |
| Zero Life events (reference) |  |  |  |  |  |  |  |  |
| One Life event | 1.29(-0.94 to 3.52) | 0 | 0.55(-1.81 to 2.90) | 15 | 0.19(-2.06 to 2.43) | 0 | 1.29(-0.94 to 3.52) | 0 |
| Two Life events | 3.04(0.40 to 5.69) | 0 | 1.18(-1.28 to 3.63) | 0 | 0.30(-2.69 to 3.30) | 0 | 3.04(0.40 to 5.69) | 0 |
| Three or More Life events | 3.07(-0.02 to 6.16) | 0 | 1.65(-1.40 to 4.70) | 0 | 0.67(-2.92 to 4.26) | 0 | 3.07(-0.02 to 6.16) | 0 |
| Arguments | 3.01(0.70 to 5.32) | 0 | 2.67(0.47 to 4.88) | 0 | 2.14(-0.19 to 4.47) | 0 | 3.01(0.70 to 5.32) | 0 |
| Bereavement | -0.04(-2.30 to 2.21) | 0 | -0.71(-2.89 to 1.46) | 0 | -0.78(-2.97 to 1.42) | 0 | -0.04(-2.30 to 2.21) | 0 |
| Debt | 2.36(0.21 to 4.51) | 0 | 0.86(-1.67 to 3.40) | 30 | 1.18(-1.26 to 3.62) | 0 | 2.36(0.21 to 4.51) | 0 |
| Divorce | 1.60(-1.14 to 4.33) | 0 | 1.48(-1.05 to 4.01) | 0 | 1.77(-2.64 to 6.18) | 45 | 1.60(-1.14 to 4.33) | 0 |
| Victim of violent crime | 3.62(-0.77 to 8.02) | 0 | 3.79(-0.25 to 7.83) | 0 | 3.57(-0.63 to 7.78) | 0 | 3.62(-0.77 to 8.02) | 0 |
| Illness or Injury | -0.16(-2.31 to 1.98) | 6 | -0.6(-2.64 to 1.43) | 0 | -0.91(-2.96 to 1.14) | 0 | -0.16(-2.31 to 1.98) | 6 |
| Legal troubles | 0.85(-2.39 to 4.08) | 0 | 0.71(-2.20 to 3.62) | 0 | 0.35(-2.67 to 3.37) | 0 | 0.85(-2.39 to 4.08) | 0 |
| Sacked/Lost job | -0.50(-5.69 to 4.69) | 58 | 0.14(-4.47 to 4.75) | 44 | -0.67(-7.07 to 5.74) | 52 | -0.50(-5.69 to 4.69) | 58 |
| ^adjusted for allocated treatment, gender, and age; *adjusted for treatment, gender, age, ethnicity, baseline BDI-II score, average anxiety duration, depression duration, and comorbid panic disorder; **‡**adjusted for treatment, gender, age, ethnicity, baseline BDI-II score, average anxiety duration, depression duration, comorbid panic disorder, social support, marital status, employment status, and financial strain. | | | | | | | | |

**Supplementary Table 19.** Results of original analyses and corresponding sensitivity analyses removing studies due to heterogeneity.

| **Prognostic Variable** | **Sample and size** | **Analysis** | **Pooled Effect Estimate** |
| --- | --- | --- | --- |
|  |  |  | **Mean difference (95%CI)** |
| Sacked/Lost job | Subgroup with first life-time depressive episode n=610 | Original analysis of association with z-score at 3-4 months post-baseline adjusted for treatment, gender, and age | 0.35(-0.45 to 1.16) |
|  |  | Analysis removing COBALT due to heterogeneity | -0.05(-0.49 to 0.38) |
|  |  | Original analysis of association with z-score at 3-4 months post-baseline adjusted for treatment, gender, age, ethnicity, baseline BDI-II score, average anxiety duration, depression duration, and comorbid panic disorder | 0.40(-0.37 to 1.18) |
|  |  | Analysis removing COBALT due to heterogeneity | 0.01(-0.39 to 0.41) |
|  |  | Original analysis of association with z-score at 3-4 months post-baseline adjusted for treatment, gender, age, ethnicity, baseline BDI-II score, average anxiety duration, depression duration, comorbid panic disorder, history of antidepressant treatment, social support, marital status, employment status, and financial strain | 0.27(-0.47 to 1.01) |
|  |  | Analysis removing COBALT due to heterogeneity | -0.06(-0.61 to 0.48) |
|  |  | Original analysis of association with z-score at 3-4 months post-baseline adjusted for treatment, gender, age, ethnicity, baseline BDI-II score, average anxiety duration, depression duration, and comorbid panic disorder | 0.40(-0.39 to 1.18) |
|  |  | Analysis removing COBALT due to heterogeneity | -0.05(-0.45 to 0.35) |
|  |  |  | **% difference (95%CI)** |
|  |  | Original analysis of association with log outcome at 3-4 months post-baseline adjusted for treatment, gender, and age | 18.65(-25.2 to 88.21) |
|  |  | Analysis removing COBALT due to heterogeneity | -3.23(-30.88 to 35.48) |
|  |  | Original analysis of association with log outcome at 3-4 months post-baseline adjusted for treatment, gender, age, ethnicity, baseline BDI-II score, average anxiety duration, depression duration, and comorbid panic disorder | 24.99(-19.15 to 93.24) |
|  |  | Analysis removing COBALT due to heterogeneity | 3.62(-24.86 to 42.89) |
|  |  | Original analysis of association with log outcome at 3-4 months post-baseline adjusted for treatment, gender, age, ethnicity, baseline BDI-II score, average anxiety duration, depression duration, and comorbid panic disorder | 24.04(-20.33 to 93.13) |
|  |  | Analysis removing COBALT due to heterogeneity | -2.37(-28.85 to 33.96) |

**Supplementary Figure 1.** Forest plots of associations between the number of life events and the z-score of depressive symptom scales at 3-4 months post-baseline independent of treatment, and other factors in Models 2, 3, and 4, on whole sample (n=2858).

**

**Supplementary Figure 2.** Forest plots of associations between individual life events and the z-score of depressive symptom scales at 3-4 months post-baseline independent of treatment, and other factors in Models 2, 3, and 4, on whole sample (n=2858).

***Supplementary* Figure 3.** Forest plots of associations between the number of life events and the z-score of depressive symptom scales at 3-4 months post-baseline independent of treatment, and other factors in Models 2, 3, and 4, on subsample (n=1910).

**Supplementary Figure 4.** Forest plots of associations between individual life events and the z-score of depressive symptom scales at 3-4 months post-baseline independent of treatment, and other factors in Models 2, 3, and 4, on subsample who had at least six months of depression at baseline (n=1910).

**Supplementary Figure 5.** Forest plots of associations between the number of life events and the z-score of depressive symptom scales at 3-4 months post-baseline independent of treatment, and other factors in Models 2, 3, and 4, on subsample with first life-time depressive episode (n=610).

**Supplementary Figure 6.** Forest plots of associations between individual life events and the z-score of depressive symptom scales at 3-4 months post-baseline independent of treatment, and other factors in Models 2, 3, and 4, on subsample with first life-time depressive episode (n=610).

## Additional References

Beck, A.T., Steer, R.A., Brown, G.K., 1996. Manual for the Beck Depression Inventory-II. Man. Beck Depress. Invent.

Buckman, J.E.J., Saunders, R., Cohen, Z.D., Clarke, K., Ambler, G., DeRubeis, R.J., Gilbody, S., Hollon, S.D., Kendrick, T., Watkins, E., White, I.R., Lewis, G., Pilling, S., 2020. What factors indicate prognosis for adults with depression in primary care? A protocol for meta-analyses of individual patient data using the Dep-GP database. Wellcome Open Res. 4, 69. https://doi.org/10.12688/wellcomeopenres.15225.3

Fisher, D.J., 2015. Two-stage individual participant data meta-analysis and generalized forest plots. Stata J. 2, 369–396.

Higgins, J.P.T., Thompson, S.G., Deeks, J.J., Altman, D.G., 2003. Measuring inconsistency in meta-analyses. Br. Med. J. 327, 557–560.

Holmes, T.H., Rahe, R.H., 1967. The social readjustment rating scale. J. Psychosom. Res. 11, 213–218.

Horton, N.J., Lipsitz, S.R., 2001. Multiple Imputation in Practice: Comparison of Software Packages for Regression Models With Missing Variables. Am. Stat. 55, 244–254. https://doi.org/10.1198/000313001317098266

Kessler, D., Lewis, G., Kaur, S., Wiles, N., King, M., Weich, S., Sharp, D.J., Araya, R., Hollinghurst, S., Peters, T.J., 2009. Therapist-delivered internet psychotherapy for depression in primary care: a randomised controlled trial. Lancet 374, 628–634. https://doi.org/10.1016/S0140-6736(09)61257-5

Kroenke, K., Spitzer, R.L., Williams, J.B.W., 2001. The PHQ-9. J. Gen. Intern. Med. 16, 606–613. https://doi.org/10.1046/j.1525-1497.2001.016009606.x

Lewis, G., Pelosi, A.J., Araya, R., Dunn, G., 1992. Measuring psychiatric disorder in the community : a standardized assessment for use by lay interviewers. Psychol. Med. 22, 465–486.

Lewis, Gemma, Duffy, L., Ades, A., Amos, R., Araya, R., Brabyn, S., Button, K.S., Churchill, R., Derrick, C., Dowrick, C., Gilbody, S., Fawsitt, C., Hollingworth, W., Jones, V., Kendrick, T., Kessler, D., Kounali, D., Khan, N., Lanham, P., Pervin, J., Peters, T.J., Riozzie, D., Salaminios, G., Thomas, L., Welton, N.J., Wiles, N., Woodhouse, R., Lewis, Glyn, 2019. The clinical effectiveness of sertraline in primary care and the role of depression severity and duration (PANDA): a pragmatic, double-blind, placebo-controlled randomised trial. The Lancet Psychiatry 6, 903–914. https://doi.org/10.1016/S2215-0366(19)30366-9

McManus, S., Bebbington, P., Jenkins, R., Brugha, T., 2016. Mental health and wellbeing in England: Adult Psychiatric Morbidity Survey 2014, NHS Digital. NHS Digital, Leeds. https://doi.org/10.1103/PhysRevB.77.235410

Morris, T.P., White, I.R., Royston, P., 2014. Tuning multiple imputation by predictive mean matching and local residual draws. BMC Med. Res. Methodol. 14, 75. https://doi.org/10.1186/1471-2288-14-75

Resche-Rigon, M., White, I.R., Bartlett, J.W., Peters, S.A.E., Thompson, S.G., 2013. Multiple imputation for handling systematically missing confounders in meta-analysis of individual participant data. Stat. Med. 32, 4890–4905. https://doi.org/10.1002/sim.5894

Royston, P., 2009. Multiple imputation of missing values: Further update of ice, with an emphasis on categorical variables. Stata J. 9, 466–477.

Royston, P., White, I.R., 2011. Multiple Imputation by Chained Equations (MICE): Implementation in Stata. J. Stat. Softw. 45, 1–20. https://doi.org/10.1002/mpr.329.Multiple

Shrier, I., Platt, R.W., 2008. Reducing bias through directed acyclic graphs. BMC Med. Res. Methodol. 8, 1–15. https://doi.org/10.1186/1471-2288-8-70

StataCorp, 2019. Stata Statistical Software: Release 16. Stata Stat. Softw. https://doi.org/10.2307/2234838

White, I.R., 2011. Multivariate Random-effects Meta-regression: Updates to Mvmeta. Stata J. Promot. Commun. Stat. Stata 11, 255–270. https://doi.org/10.1177/1536867X1101100206
